# Deep Learning-Predicted Dihydroartemisinin Rescues Osteoporosis by Maintaining Mesenchymal Stem Cell Stemness through Activating Histone 3 Lys 9 Acetylation

**DOI:** 10.1021/acscentsci.3c00794

**Published:** 2023-10-18

**Authors:** Ruoxi Wang, Yu Wang, Yuting Niu, Danqing He, Shanshan Jin, Zixin Li, Lisha Zhu, Liyuan Chen, Xiaolan Wu, Chengye Ding, Tianhao Wu, Xinmeng Shi, He Zhang, Chang Li, Xin Wang, Zhengwei Xie, Weiran Li, Yan Liu

**Affiliations:** † Laboratory of Biomimetic Nanomaterials, Department of Orthodontics & National Center for Stomatology & National Clinical Research Center for Oral Diseases & National Engineering Laboratory for Digital and Material Technology of Stomatology & Beijing Key Laboratory of Digital Stomatology & Research Center of Engineering and Technology for Computerized Dentistry Ministry of Health & NMPA Key Laboratory for Dental Materials & Translational Research Center for Orocraniofacial Stem Cells and Systemic Health, Peking University School and Hospital for Stomatology, Beijing 100081, China; ‡ Central Laboratory, National Center for Stomatology & National Clinical Research Center for Oral Diseases & National Engineering Laboratory for Digital and Material Technology of Stomatology & Beijing Key Laboratory of Digital Stomatology & Research Center of Engineering and Technology for Computerized Dentistry Ministry of Health & NMPA Key Laboratory for Dental Materials & Translational Research Center for Orocraniofacial Stem Cells and Systemic Health, Central Laboratory, Peking University School and Hospital for Stomatology, Beijing 100081, China; Δ Peking University International Cancer Institute, Health Science Center, Peking University, Beijing 100083, China

## Abstract

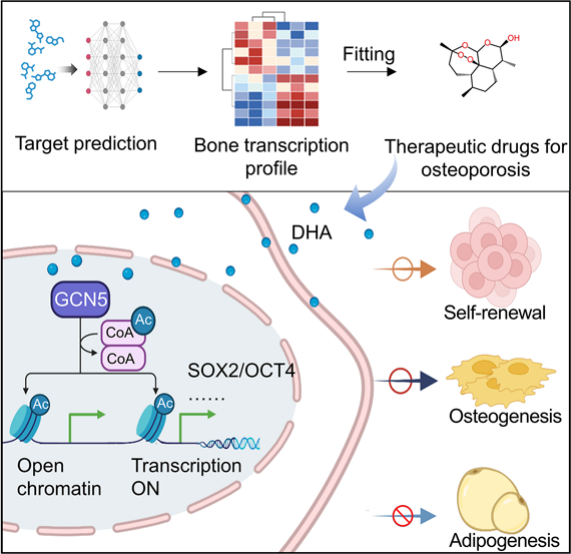

Maintaining the stemness of bone marrow mesenchymal stem cells (BMMSCs) is crucial for bone homeostasis and regeneration. However, *in vitro* expansion and bone diseases impair BMMSC stemness, limiting its functionality in bone tissue engineering. Using a deep learning-based efficacy prediction system and bone tissue sequencing, we identify a natural small-molecule compound, dihydroartemisinin (DHA), that maintains BMMSC stemness and enhances bone regeneration. During long-term *in vitro* expansion, DHA preserves BMMSC stemness characteristics, including its self-renewal ability and unbiased differentiation. In an osteoporosis mouse model, oral administration of DHA restores the femur trabecular structure, bone density, and BMMSC stemness *in situ*. Mechanistically, DHA maintains BMMSC stemness by promoting histone 3 lysine 9 acetylation via GCN5 activation both *in vivo* and *in vitro*. Furthermore, the bone-targeted delivery of DHA by mesoporous silica nanoparticles improves its therapeutic efficacy in osteoporosis. Collectively, DHA could be a promising therapeutic agent for treating osteoporosis by maintaining BMMSC stemness.

## Abstract 2

Dihydroartemisinin, as predicted by deep learning, rescues osteoporosis by preserving the stemness of bone marrow mesenchymal stem cells through the activation of Histone 3 Lys 9 acetylation.

## Introduction

Osteoporosis is a degenerative disease that affects the skeletal system and is characterized by the loss of bone density and destruction of the bone microstructure.^1^ The pathogenesis of osteoporosis involves disruption of the balance between bone formation and resorption,^2^ which is caused by excessive bone resorption by osteoclasts and insufficient bone repair and reconstruction due to reduced osteoblast functions.^3^ Bone marrow-derived mesenchymal stem cells (BMMSCs), which are the precursors of osteoblasts,^4^ play a crucial role in osteoporosis. BMMSCs maintain a constant flow of functional osteoblasts by committed differentiation and a local population through steady proliferation and refreshment, which together constitute the stemness of BMMSCs under physiological motion.^5,6^ However, recent studies have established that during osteoporosis, BMMSCs exhibit biased differentiation trends toward adipocytes and diminished regenerative potential.^7,8^

Current mainstay drugs for osteoporosis, such as estrogens and bisphosphonates, mainly target hormone deficiency or bone resorption but do not directly restore the stemness and vitality of BMMSCs.^2,9^ Because BMMSCs provide a continuous supply of osteoblasts for bone repair, it is critical to find ways to restore their functions. Gene editing, cytokines, medicated additives, and physical/chemical stimulation have been shown to promote the maintenance of BMMSC stemness in bone tissue engineering.^10−13^ Among these approaches, small molecules stand out because of their low cost, widespread availability, and biocompatibility.^14^

Deep learning has emerged as a promising tool to accelerate drug development. This allows the analysis of vast amounts of transcriptional data to identify potential drug targets and predict the efficacy of new drugs.^15^ Gene set enrichment analysis (GSEA) implied in the Connectivity Map (CMap) was used to identify drugs with potential therapeutic effects.^16−18^ In our recent studies, we trained a deep learning algorithm based on L1000 drug-transcriptome data to predict cellular responses with drug treatment and eventually accurately predicted the efficacies of new drugs by comparing the changes in gene expression profiles of diseased and drug-treated cells.^17,19^ This deep learning-based efficacy prediction system (DLEPS) has already been successful in discovering new drugs for a range of diseases, including obesity, hyperuricemia, and NASH.^17^

In this study, we employed DLEPS analysis for efficacy scores based on the differentially expressed genes (DEGs) in the bone tissues of neonatal mice compared to adult mice.^17,20^ From the top-ranked candidates, we identified dihydroartemisinin (DHA), a traditional Chinese herbal extract that can promote BMMSC stemness, which is beneficial for establishing healthier bone homeostasis.^21^ This prediction was confirmed by the enhanced proliferation ability and unbiased differentiation potential during *in vitro* long-term serial passaging. Systemic administration and bone-targeted delivery of DHA by mesoporous silica nanoparticles to bone tissues effectively inhibited bone loss and maintained endogenous BMMSC functions in mice with ovariectomy-induced osteoporosis. Mechanistically, DHA achieved its stemness-maintaining capacity by upregulating the GCN5-H3K9ac axis at the epigenetic level, which allowed the downstream expression of stemness-related genes.^21^ In short, our research has completed the entire process of discovery, functional validation, mechanism exploration, and bone-targeted delivery of the small molecule drug DHA. This comprehensive study has effectively demonstrated that DHA holds immense potential as a therapeutic agent for the treatment of osteoporosis.

## Results

### A Deep Learning-Predicted DHA Enhances BMMSC Stemness and Osteogenic Differentiation

**Figure 1:**
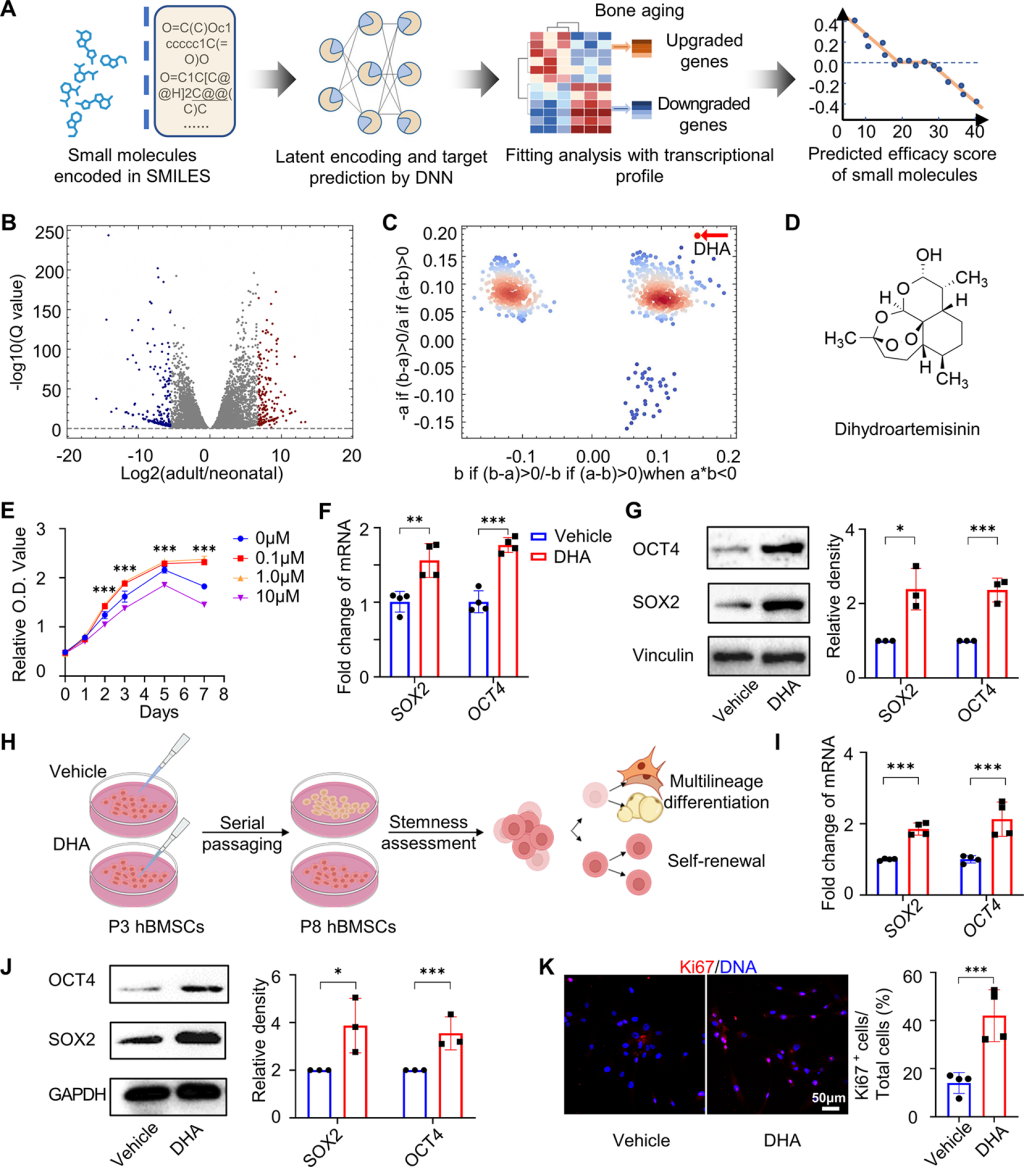
A deep-learning predicted DHA maintains stemness of hBMMSCs both in the early passage and during long-term passaging *in vitro*. (A) Schematic illustration of the drug screening process via DLEPS. (B) A volcano map of transcriptional profile alterations between neonatal and adult mouse bones. The blue dots represent downregulated genes in adult mouse femora compared to neonatal ones (*n* = 3). (C) The enrichment scores of small molecules. The red dots represent the score distribution of DHA. (D) Molecule structure of DHA. (E) CCK8 assay of the optimum concentrations to treat hBMMSCs (*n* = 6). (F) RT-qPCR of stemness-related markers *SOX2* and *OCT4* in vehicle- and DHA-treated hBMMSCs (*n* = 4). (G) Western blotting of SOX2 and OCT4 in vehicle- and DHA-treated hBMMSCs (*n* = 3). (H) Schematic showing the serial passage of hBMMSCs. (I) RT-qPCR of *SOX2* and *OCT4* in hBMMSCs after vehicle and DHA treatment for 5 generations, respectively (*n* = 4). (J) Western blotting of SOX2 and OCT4 in hBMMSCs after vehicle and DHA treatment for 5 generations, respectively (*n* = 3). (K) Immunofluorescence staining and semiquantitative analysis of Ki67 in vehicle- and DHA-treated hBMMSCs after 5 generations (*n* = 3). Data were represented as mean ± SD, and the *P* values were calculated by a two-tailed Student’s *t*-test. Statistical significance was defined as ***P < 0.001, **P < 0.01, and *P < 0.05 between the vehicle group and the DHA group.

BMMSCs often suffer from stemness loss and senescent hypofunction during *in vitro* passaging, leading to impaired proliferation, biased differentiation into adipocytes, and limited therapeutic efficacy for bone regeneration.^22^ To overcome these limitations and improve the availability of functional BMMSCs, we first measured the DEGs of femur tissues between neonatal (postnatal day 1) and adult mice (6–8 weeks old) by RNA sequencing since the stemness of mesenchymal stem cells gradually diminished during tissue maturation, and then utilized DLEPS to predict the small molecules that can reverse these DEGs to enhance BMMSC stemness (Figure 1A,B).^23^ The ranks of the DEGs in the 12328 genes, whose activity can be predicted in DLEPS, were used to calculate a bone score (Figure 1B). For the user-defined library, we chose TargetMol, an FDA-approved library containing 961 compounds. The enrichment scores of up- and downregulated genes are plotted as *x* and *y* coordinates in Figure 1C (see Methods for details). Lastly, we selected the intersection of the top-ranked scores *|a – b|* (10 molecules in total) to be tested in the cell culture or directly in the animal models (Table S1). Among the candidates, DHA, a traditional Chinese herbal extract, was selected for its good pharmacological properties (Figure 1C,D). Although studies have demonstrated the functional roles of DHA as an antimalarial, antitumor, and immunoregulatory molecule, reports on its activity in the regulation of stemness are currently lacking. To determine whether DHA rescues the phenotypic characteristics of BMMSCs, we used a replicative cellular senescence model that has been widely utilized to investigate cell stemness. A cell counting kit-8 (CCK8) assay showed that DHA at a concentration of 0.1 μM possessed the optimal biocompatibility to human BMMSCs (hBMMSCs) (Figure 1E). Western blotting confirmed that hBMMSCs treated with 0.1 μM DHA showed a remarkably higher expression of SOX2 and OCT4 (the encoding gene of OCT4 is also known as *POU5F1*) (Figure 1F,G). Next, DHA at a concentration of 0.1 μM was added to the growth medium of hBMMSCs in each passage from passage 3 (P3) to passage 8 (P8). The control group was added with the same amount of dimethyl sulfoxide (DMSO), the vehicle of DHA (Figure 1H). Quantitative real-time reverse transcription polymerase chain reaction (RT-qPCR) and Western blotting results revealed that DHA maintained hBMMSC stemness by enhancing *SOX2* and *OCT4* expression during the 5-passage expansion (Figure 1I,J). Moreover, Ki67 immunofluorescent staining indicated that DHA-treated hBMMSCs possessed a better proliferation capacity (Figure 1K).

**Figure 2:**
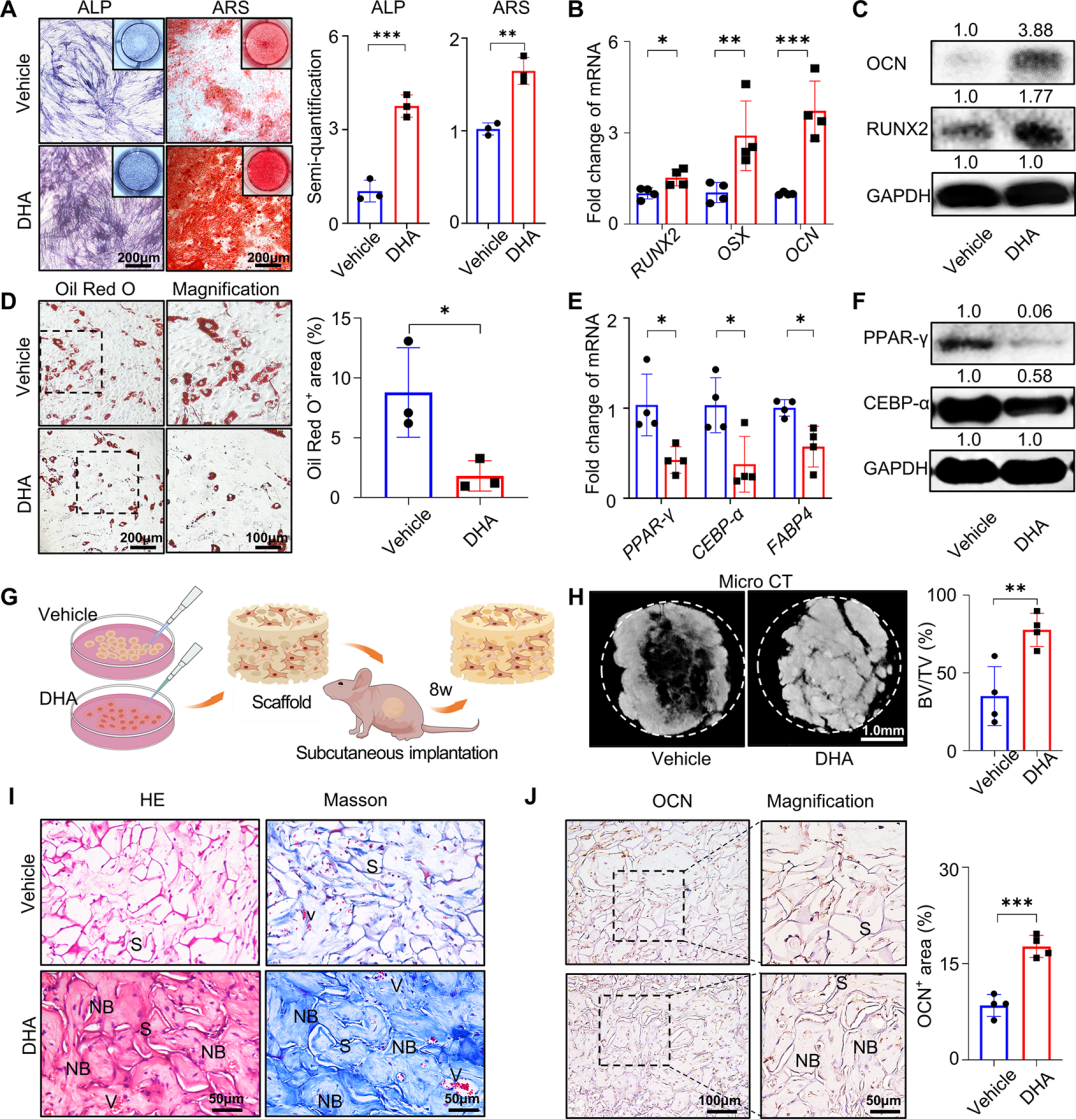
DHA maintains unbiased differentiation potentials of hBMMSCs during long-term passaging. (A) ALP and ARS stainings of hBMMSCs treated with vehicle or DHA for 5 generations followed by osteogenic induction (*n* = 3). (B) RT-qPCR of osteogenesis-related genes *RUNX2*, *OSX*, and *OCN* in hBMMSCs treated with vehicle or DHA for 5 generations followed by osteogenic induction (*n* = 4). (C) Western blotting of osteogenesis-related proteins RUNX2 and OCN in hBMMSCs treated with vehicle or DHA for 5 generations followed by osteogenic induction (*n* = 3). (D) Oil red O staining of hBMMSCs treated with vehicle or DHA for 5 generations followed by adipogenic induction (*n* = 3). (E) RT-qPCR of adipogenesis-related genes *PPAR-γ*, *CEBP-α*, and *FABP-4* in hBMMSCs treated with vehicle or DHA for 5 generations followed by adipogenic induction (*n* = 4). (F) Western blotting of adipogenesis-related proteins PPAR-γ and CEBP-α in hBMMSCs treated with vehicle or DHA for 5 generations followed by adipogenic induction. (G) Schematic showing the subcutaneous implantation of the mineralized collagen scaffolds loaded with the vehicle- or DHA-treated hBMMSCs in nude mice. (H) Micro CT of reconstructed 3D images of mineralized implants and quantified bone volume fraction (*n* = 4). (I) HE and Masson’s trichrome stainings of representative regions of the mineralized implants. S: scaffold; NB: new bone; V: blood vessel. (J) Immunohistochemical staining of OCN and semiquantitation of positive cells (*n* = 4). Data were represented as mean ± SD, and the *P* values were calculated by a two-tailed Student’s *t*-test. Statistical significance was defined as ***P < 0.001, **P < 0.01, and *P < 0.05 between the vehicle group and the DHA group.

Since unbiased differentiation is an essential characteristic of mesenchymal stem cells,^24^ we evaluated whether sustained exposure to DHA helped maintain the osteogenic differentiation potential of hBMMSCs and inhibited their adipogenic differentiation potential after prolonged passages. Before differentiation induction, we stopped DHA treatment of hBMMSCs at P8 and then transferred the vehicle- and DHA-treated passaged hBMMSCs into a differentiation medium without DHA. DHA-treated passaged hBMMSCs demonstrated a higher osteogenic potential than the control group, as evidenced by a larger mineralization area in alizarin red S staining (ARS) and stronger alkaline phosphatase activity (Figure 2A). In contrast, DHA-treated passaged hBMMSCs showed a lower adipogenic tendency, indicated by less lipid droplet formation in Oil red O staining (Figure 2D). Therein, biased differentiation can be induced by the imbalance between runt-related transcription factor 2 (*RUNX2*) and the peroxisome proliferator-activated receptor γ (*PPAR-γ*) pathway, so we further confirmed the hBMMSC fate determination trend by RT-qPCR and Western blotting. DHA-treated passaged hBMMSCs showed a higher expression of osteogenic markers including *RUNX2*, *Osterix* (*OSX*), and *osteocalcin* (*OCN*) after osteogenic induction (Figure 2B,C), while high expression of adipocytic markers including *PPAR-γ*, *CCAAT-enhancer-binding proteins α* (*CEBP-α*), and *fatty-acid-binding protein 4* (*FABP4*) was detected in the vehicle control group (Figure 2E,F).

To test the osteogenic ability of the expanded stem cells *in vivo*, we subcutaneously implanted mineralized collagen scaffolds seeded with DHA-treated passaged hBMMSCs into nude mice (Figure 2G).^25^ After 8 weeks of implantation, microcomputed tomography (micro CT) images revealed high-density bone-like tissue in both implant groups, while the ratio of bone volume to total volume in the DHA group (77.67 ± 10.71%) was obviously higher than that in the control group (35.10 ± 18.96%) (Figure 2H). Massive new bone tissues were formed with orderly arranged collagen fibers in the DHA group, as evidenced by HE and Masson staining. Moreover, immunohistochemical staining revealed that OCN, an osteoblast-specific secreted protein, was highly expressed in bone lacunae and around calcified regions. In contrast, patches of mineralization areas and disordered fibers were distributed sporadically in the vehicle group, showing a structure distinct from that of natural bone tissues (Figure 2I,J).

### Systemic Delivery of DHA Rescues OVX-Induced Osteoporosis in Mice

**Figure 3:**
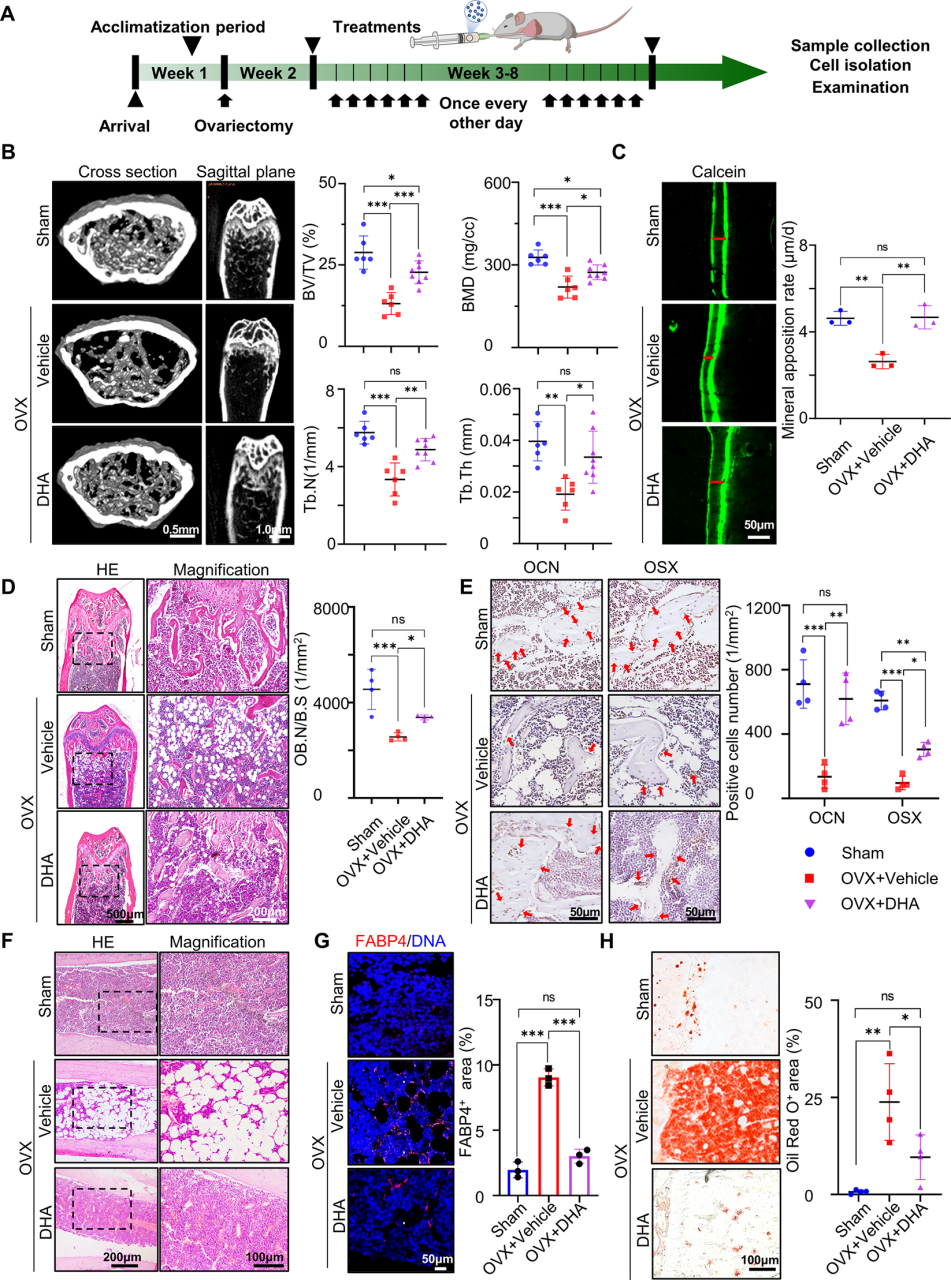
Long-term oral administration of DHA rescues bone loss in osteoporotic mice. (A) Schematic illustration of the design of animal experiments. (B) Micro CT images of reconstructed 3D images and bone morphometric parameters of the trabecular bone from different groups (*n* = 6). (C) Sequential fluorescent labeling indicating bone mineral apposition width (red line) during 7 days and semiquantification (*n* = 3). (D) HE staining of femora from different groups and semiquantitative analysis of osteoblast number per bone surface (OB.N/B.S) (*n* = 4). (E) Immunohistochemical staining of OCN^+^ and OSX^+^ cells (red arrow) in femur bone marrow after DHA treatment, respectively, and semiquantification (*n* = 4). (F) HE staining of tibiae after DHA treatment. (G) Immunofluorescence staining of FABP4^+^ cells in tibiae and semiquantification (*n* = 3). (H) Oil red O staining of proximal tibiae and semiquantitative analysis of the positive area (*n* = 4). Data were represented as mean ± SD, the *P* values were calculated by one-way ANOVA with Tukey as a posthoc test, and the statistical significance was defined as ***P < 0.001, **P < 0.01 and *P < 0.05 among different groups.

The basic mechanism of osteoporosis involves an imbalance between impaired bone formation caused by osteoblast hypofunction and excessive bone resorption induced by osteoclast hyperfunction. However, bone mass loss and fat accumulation are strongly relevant to the biased differentiation of BMMSCs toward the adipogenic lineage during the pathological process of osteoporosis.^26^ Therefore, we established an ovariectomized (OVX) mouse model of osteoporosis to test the therapeutic effects of DHA on skeletal diseases. Mice in the experimental group were intragastrically administered with 10 mg/kg DHA every other day for 1 week postsurgery (OVX + DHA group). DMSO was used as a vehicle for the better dissolution of DHA and the same amount of vehicle was administered to osteoporotic mice by gavage as the control group (OVX + Vehicle group). Mice that underwent sham surgery were used as blank controls (sham group). Organs were harvested 8 weeks later (Figure 3A).

According to the micro CT imaging, mice with ovariectomy exhibited significant trabecular loss in distal metaphysis compared to the sham group, with a decrease in bone volume/total volume (BV/TV) from 28.84 ± 5.11% (sham group) to 13.14 ± 3.36% (OVX group), trabecular thickness (Tb.Th) from 0.049 ± 0.004 mm (sham group) to 0.040 ± 0.004 mm (OVX + vehicle group), trabecular number (Tb.N) from 5.75 ± 0.58/mm (sham group) to 3.34 ± 0.85/mm (OVX + vehicle group), and a decrease in bone mineral density (BMD) from 327.27 ± 27.34 mg/cm^3^ (sham group) to 219.72 ± 39.75 mg/cm^3^ (OVX + vehicle group). DHA treatment obviously reversed bone loss in the distal femur, evidenced by increased BV/TV to 22.77 ± 3.50%, Tb.Th to 0.047 ± 0.005 mm, Tb.N to 4.88 ± 0.58/mm, and BMD to 273.18 ± 26.85/mm (Figure 3B). The results of HE staining were consistent with those of the radioautographic imaging. The marrow cavity of femora from the mice in the OVX + vehicle group was occupied with vacuoles, with sparse trabecular distribution inside. In contrast, well-structured dense trabeculae lined with osteoblasts were observed in the femora from the mice in the OVX + DHA group, similar to those in the sham group. Osteoblast number per bone surface (OB.N/B.S) in the OVX + DHA group was significantly higher than that in the OVX + vehicle group (Figure 3D). Similar histological features were observed in the tibia by HE staining (Figure 3F). The mineral apposition rate on the femur surface was measured by using calcein double labeling. In the OVX + DHA group, it reached 32.7 ± 2.7 mm/day, showing no statistical differences with that in the sham group (32.4 ± 2.3 mm/day), whereas it was faster than that of the OVX + vehicle group (18.4 ± 2.3 mm/day) (Figure 3C). Immunohistochemical images also showed higher expression levels of the osteogenic differentiation markers OCN and OSX in femora from DHA-treated osteoporotic mice (Figure 3E), which corroborated the promotive effects of DHA on bone conservation and formation. Apart from the femora, DHA also showed a protective effect on periodontal bone tissue. The height loss of the first molar mesial alveolar bone increased to 0.20 ± 0.04 mm in OVX mice, which was as twice as that in the sham group, while it decreased to 0.14 ± 0.02 mm in DHA-treated mice (Figure S1).

The fat distribution range was also determined in the tibia, as biased differentiation of BMMSCs could underlie fat accumulation in osteoporotic bone marrow.^27^ The Fabp4 positive area was mainly concentrated around the vacuoles in the mainstay, and the mesial metaphysis of the tibiae was filled with lipid droplets in OVX mice. However, the Fabp4 expression level and Oil red O-positive area was significantly reduced after DHA treatment (Figure 3G,H). Notably, no signs of toxicity were detected by HE staining of the liver and kidneys from DHA-treated mice (Figure S2).

### DHA Maintains Endogenous Mouse BMMSC Stemness and Unbiased Differentiation Potentials in Situ

**Figure 4:**
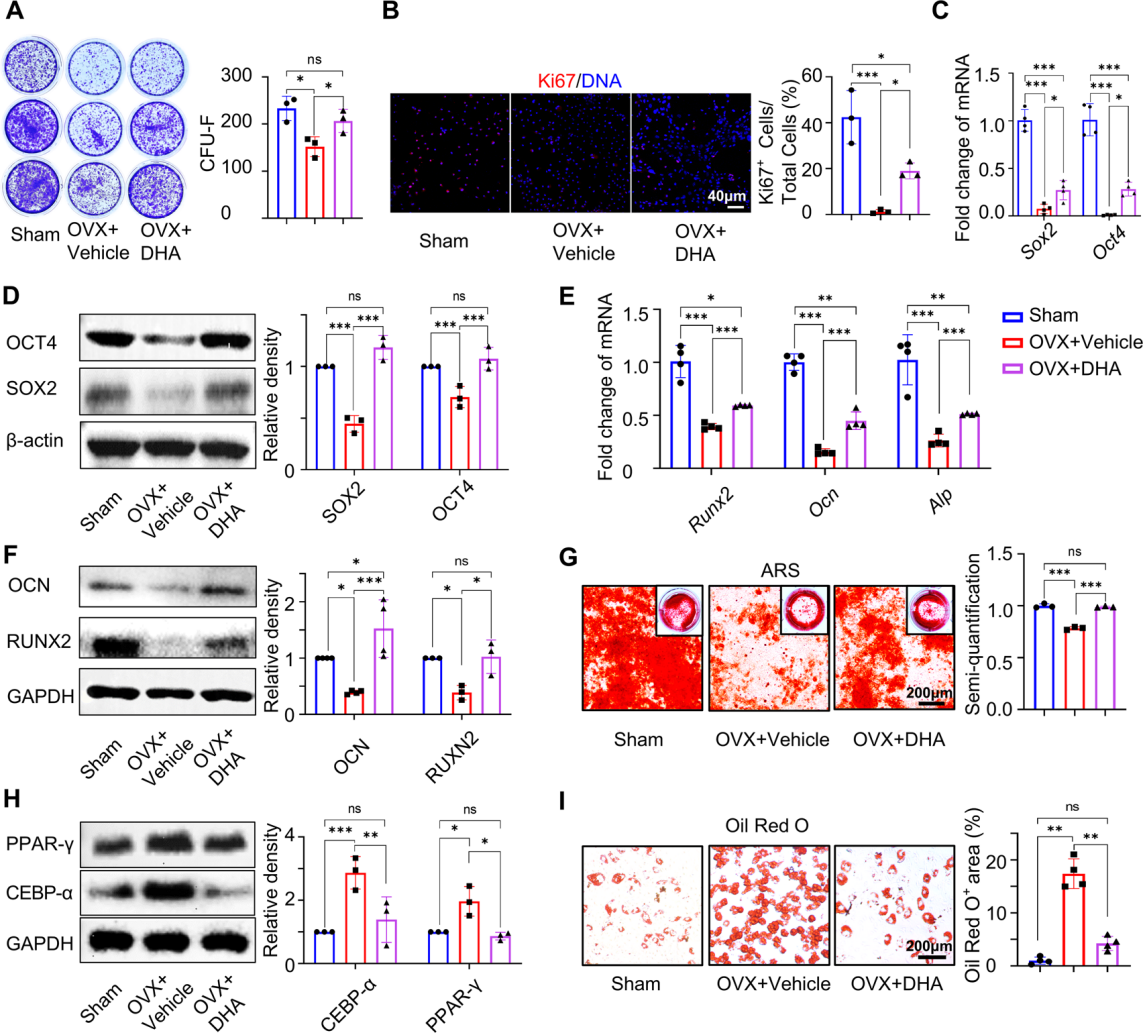
DHA improves stemness and unbiased differentiation potentials of endogenous mBMMSCs from osteoporotic mice. (A) CFU assay of mBMMSCs isolated from normal mice (sham group), vehicle-treated OVX mice (OVX + vehicle group), and DHA-treated OVX mice (OVX + DHA group) (*n* = 3). (B) Immunofluorescence staining and semiquantitative analysis of Ki67^+^ mBMMSCs isolated from the sham, OVX + vehicle, and OVX + DHA groups (*n* = 3). (C) RT-qPCR of *Sox2* and *Oct4* in mBMMSCs isolated from the sham, OVX + vehicle, and OVX + DHA groups (*n* = 4). (D) Western blotting of OCT4 and SOX2 in mBMMSCs isolated from the sham, OVX + vehicle, and OVX + DHA groups (*n* = 3). (E) RT-qPCR of *Runx2, Ocn*, and *Alp* in mBMMSCs isolated from the sham, OVX + vehicle, and OVX + DHA groups with osteogenic induction (*n* = 4). (F) Western blotting of OCN and RUNX2 in mBMMSCs isolated from the sham, OVX + vehicle, and OVX + DHA groups with osteogenic induction (*n* = 3). (G) ARS staining and semiquantification of mBMMSCs isolated from the sham, OVX + vehicle, and OVX + DHA groups with osteogenic induction (*n* = 3). (H) Western blotting of PPAR-γ and CEBP-α in mBMMSCs isolated from the sham, OVX + vehicle, and OVX + DHA groups with adipogenic induction (*n* = 3). (I) Oil red O staining and semiquantification of mBMMSCs isolated from the sham, OVX + vehicle, and OVX + DHA groups with adipogenic induction (*n* = 4). Data were represented as mean ± SD, the *P* values were calculated by one-way ANOVA with Tukey as a posthoc test, and the statistical significance was defined as ***P < 0.001, **P < 0.01, and *P < 0.05 among different groups.

BMMSCs, the precursors of the osteoblasts lineage, occupy one end of the balance of bone homeostasis.^28^ In the ongoing process of bone remodeling and repair, cytokines and other chemical factors previously present in the bone tissues are released into the bone marrow microenvironment after bone resorption, which then recruits and differentiates BMMSCs to the niche for subsequent bone reconstruction.^29^ Thus, BMMSC stemness *in situ* plays an essential role in maintaining bone mass during osteoporosis.^28^ To investigate how DHA rescues OVX-induced osteoporosis, mouse BMMSCs (mBMMSCs) were extracted from the DHA-treated OVX mouse model, and their biological functions, including stemness and osteogenic differentiation capacities, were tested. OVX significantly reduced the colony forming ability, Ki67 expression level, and stemness marker expression of mBMMSCs (Figure 4A–D). However, mBMMSCs derived from the DHA-treated mice displayed elevated colony numbers in the colony-forming unit (CFU) assay and increased Ki67 positive cells compared with the OVX + vehicle group. The expression of the stemness markers, *Sox2* and *Oct4* was also enhanced, as indicated by RT-qPCR and Western blotting (Figure 4A–D). ARS staining revealed that OVX inhibited mBMMSCs’ mineralized nodule formation compared to the sham group, whereas DHA treatment almost restored their osteogenic capacity (Figure 4G), which was also emphasized by the enhanced expression of osteogenic markers in RT-qPCR and Western blotting (Figure 4E, F). In contrast, the biased adipogenic differentiation tendency of mBMMSCs from OVX mice was increased. Western blotting showed that the expression of adipogenic-related markers CEBP-α and PPAR-γ in mBMMSCs from the OVX + vehicle group was much higher than that in the sham group, whereas this tendency could be reversed by DHA treatment (Figure 4H). Moreover, the percentage of the Oil red O-positive area in the OVX group reached 17.79 ± 2.79%, which was 16 times higher than that in the sham group. Interestingly, BMMSCs from the OVX + DHA group displayed a reduced positive area (4.23 ± 1.33%), close to that in the sham group (Figure 4I). In general, oral administration of DHA rescued endogenous mBMMSC stemness in OVX mice, while correcting the biased differentiation inclination from adipogenesis to osteogenesis.

### DHA Enhances BMMSC Stemness through Upregulating Histone 3 Lys 9 Acetylation via GCN5

Epigenetic regulation plays a crucial role in the modulation of mesenchymal stem cell functions and bone remodeling.^30^ The chromatin structure and corresponding transcriptional activity of genes change dynamically during different biological processes, including DNA methylation, histone modifications, chromatin remodeling, and noncoding RNA modulation, which are the major epigenetic mechanisms involved.^31^ The declination in biological activity and dysfunction during long-term cell expansion are no exceptions to epigenetically regulated processes.^32,33^ One major signature of cellular senescence is low transcriptional activity due to global hypoacetylation of histone lysines,^34^ which has also been verified in hBMMSCs from different passages (Figure S3). Correspondingly, histone deacetylase (HDAC) and histone acetyltransferase (HAT) levels in the bone tissues of osteoporotic mice were proven to be different from those in normal mice.^35,36^ Moreover, artemisinin, a small molecule with a chemical structure similar to DHA, was reported to upregulate the acetylation of the ninth lysine in histone 3 (H3K9) via HDACs in rat neuronal cells.^37^

**Figure 5:**
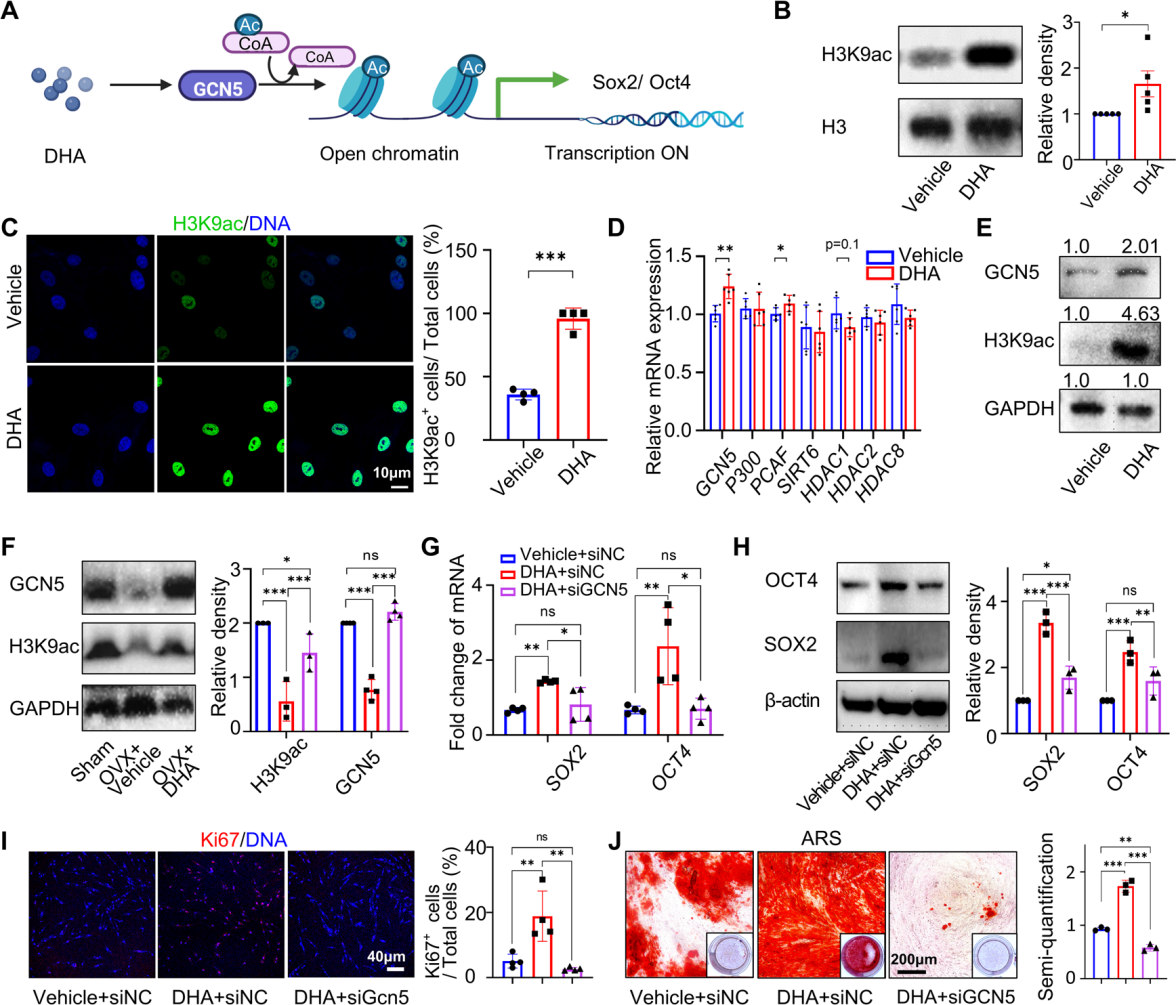
DHA enhances BMMSC stemness by histone modification. (A) Schematic showing that DHA enhances BMMSC stemness by histone acetylation. The scheme was created by BioRender (https://www.biorender.com). (B, C) Western blotting (B) and confocal microscopy (C) of H3K9ac in vehicle- and DHA-treated hBMMSCs after 5 generations (*n* = 4). (D) RT-qPCR of *GCN5*, *P300*, *PCAF*, *SIRT6*, *HDAC1*, *HDAC2*, and *HDAC8* in vehicle- and DHA-treated hBMMSCs after 5 generations (*n* = 6). (E) Western blotting of H3K9ac and GCN5 in hBMMSCs treated by vehicle and DHA for five passages (*n* = 3). (F) Western blotting of H3K9ac and GCN5 in mBMMSCs from mice with different treatments (*n* = 3). (G) RT-qPCR of *SOX2* and *OCT4* in hBMMSCs treated with vehicle or DHA for 5 generations followed by knocking down GCN5 (*n* = 4). (H) Western blotting of SOX2 and OCT4 in hBMMSCs treated with vehicle or DHA for 5 generations followed by knockdown of GCN5 (*n* = 3). (I) Immunofluorescence staining and semiquantification of Ki67 in hBMMSCs treated with vehicle or DHA for 5 generations followed by knocking down GCN5 (*n* = 4). (J) ARS staining of hBMMSCs treated with vehicle or DHA for 5 generations followed by GCN5 knockdown and osteogenic induction for 21 days and semiquantification (*n* = 3). Data were represented as mean ± SD, and the *P* values were calculated by two-tailed Student’s *t*-test for (B–D) and (I), while by one-way ANOVA with Tukey as a posthoc test for d and j–m. Statistical significance was defined as ***P < 0.001, **P < 0.01, and *P < 0.05 between the control group and the DHA-treated group.

Therefore, we hypothesized that DHA may modulate the acetylation levels of BMMSCs by influencing the activity of acetylation-related enzymes, consequently exerting regulatory control over the cellular fate (Figure 5A). To verify this hypothesis, we first tested the H3K9 acetylation (H3K9ac) level of long-term cultured hBMMSCs with nucleoproteins using Western blotting and immunofluorescence analyses, confirming the acetylation-upregulating effect of DHA during cell expansion (Figure 5B, C). To screen for the candidate upstream genes that DHA acts on, we performed RT-qPCR to test the expression levels of the 7 H3K9 acetylation-related HATs/HDACs including *GCN5*, *P200*, *PCAF*, *SIRT6*, *HDAC1*, *HDAC2*, and *HDAC8*.^38^ Among the three HATs and the four HDACs, the expression of GCN5 showed the most significant change, which was upregulated by 1.5-fold after DHA treatment (Figure 5D). Western blotting confirmed the upregulation of the GCN5 protein in DHA-treated hBMMSCs (Figure 5E). Accordingly, GCN5 protein expression and H3K9 acetylation levels in osteoporosis-derived BMMSCs were also elevated, consistent with results *in vitro* (Figure 5F).

To further elucidate whether DHA upregulates stemness markers via the GCN5-H3K9ac axis, we performed loss-of-function assays by knocking down *GCN5* after long-term DHA treatment. The siRNA efficiency was confirmed by RT-qPCR and Western blotting (Figure S4). DHA promoted the gene expression of *SOX2* and *OCT4* in hBMMSCs in P8, whereas the followed *GCN5* knockdown attenuated stemness marker expression, proliferation or osteogenic ability (Figure 5G–J). Similarly, DHA failed to upregulate stemness markers in GCN5-knockdown cells, as evidenced by RT-qPCR and Western blotting, indicating a core effect of GCN5 (Figure S5). Together, DHA maintains BMMSC stemness through upregulation of Histone 3 Lys 9 acetylation via GCN5 (Figure 5A).

### Bone-Targeted Delivery of DHA by MSN-ALN Nanospheres Promotes Bone Formation under OVX-Induced Osteoporosis

**Figure 6:**
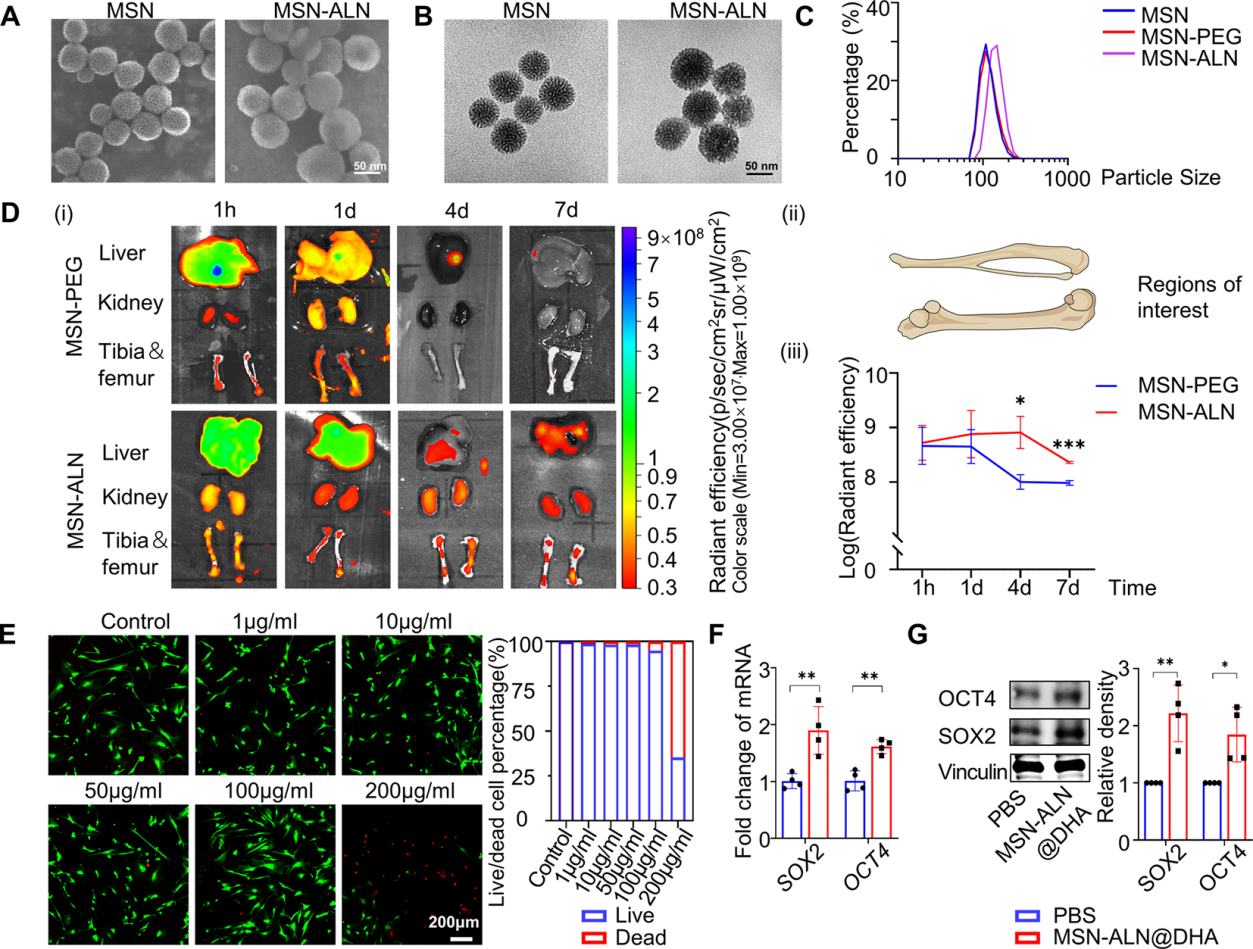
MSN-ALN nanospheres for bone-targeted delivery of DHA. (A, B) SEM (A) and TEM (B) images showing the nanostructure of MSNs and ALN-modified MSNs (MSN-ALNs). (C) Size distribution curves of MSNs, MSN-ALNs, and MSN-PEGs in PBS. (D) Representative *ex vivo* images of organs and bones (i), and quantified accumulation in bones (ii–iii) at varying intervals after tail vein injection of MSN-PEGs and MSN-ALNs in mice (*n* = 4). (E) Live and dead staining and semiquantification of hBMMSCs after incubating with MSN-ALNs for 24 h (live: green, dead: red, *n* = 4). (F) RT-qPCR of *SOX2* and *OCT4* in PBS- and MSN-ALN@DHA-treated hBMMSCs (*n* = 4). (G) Western blotting of SOX2 and OCT4 in PBS- and MSN-ALN@DHA-treated hBMMSCs (*n* = 4). Data were represented as mean ± SD, and the *P* values were calculated by two-tailed Student’s *t*-test. Statistical significance was defined as ***P < 0.001, **P < 0.01, and *P < 0.05 between the control group and the DHA-treated group.

To improve the therapeutic efficiency of DHA in osteoporosis, mesoporous silica nanoparticles (MSNs) conjugated with bone-targeting alendronate (ALN) were designed to deliver DHA.^39,40^ The MSNs were fabricated using a modified one-pot biphase stratification method.^41^ After the amine functionalization of MSN (MSN-NH_2_), *N*-hydroxyl succinimide-PEG2k-carboxyl (NHS-PEG2k-COOH) was cross-linked to fabricate MSN-PEG via click-chemistry between the NHS and amine groups.^42^ Finally, ALN, with an amine group, was covalently conjugated to MSN-PEG, with a carboxylic group, in the presence of carbodiimide (Figure S6A). Scanning electron microscopy (SEM), transmission electron microscopy (TEM), and dynamic light scattering (DLS) were used to display the morphological features of MSNs, MSN-ALNs, and key intermediate products. In general, MSNs and MSN-ALNs were monodispersed, walnut kernel-shaped porous nanoparticles with diameters of approximately 100 nm and mesopores of 2 nm, in a homogeneous dispersion state when suspended in PBS (Figure 6A–C). The intermediate products MSN-NH_2_ and MSN-PEG, which shared similar morphological features with MSNs, only possessed larger diameters (Figure S6B–D). In addition, MSN-ALNs could be gradually biodegraded in saline at 37 °C, evidenced by deformation and distortion during 7 days in TEM imaging (Figure S6E).

ALN, a traditional anti-osteoporosis drug with a high affinity for the bone surface,^43^ was used for bone-targeted delivery to increase the local DHA concentration in the bone microenvironments. We labeled the MSN-ALN and the MSN-PEG with rhodamine to confirm the bone-targeting efficacy of ALN and determined the fluorescence intensity distribution at 1 h, 1 day, 4 days, and 7 days after intravenous injection.^44^ Caliper IVIS Lumina analogue images demonstrated that there were no significant differences in the fluorescence signal distribution at 1 h and 1 day postinjection. Nevertheless, MSN-ALN retention in the femora and tibiae was elongated to 7 days, longer than 4 days in the MSN-PEG group (Figure 6D). In contrast, the fluorescence signal decreased over time in the liver and kidneys, indicating a metabolic process that meets the physiological rationale *in vivo* (Figure 6D).

The large pore volume of MSN enabled a sufficient DHA loading and controlled drug release. According to a previous study, the cargo-loading ratio could reach more than 50% in MSNs.^39^ Hence, DHA was loaded into the MSN-ALNs at different ratios using the rotary evaporation method. When the DHA loading ratio in MSN-ALNs ranged from 0 mg g^–1^ to 333 mg g^–1^, no DHA crystals remained, as detected by SEM (Figure S6F), indicating a complete load of DHA. And 333 mg g^–1^ was chosen as the optimal loading ratio, which was supported by a previous study.^39^ Thus, 0.3 μM MSN-ALN loaded with DHA was required in the culture medium to allow DHA to reach the effective dose, which did not reach the cytotoxicity threshold of MSN-ALN according to live and dead staining results (Figure 6E). The following RT-qPCR and Western blotting experiments also revealed that 0.3 μM MSN-ALN@DHA promoted stemness marker expression during hBMMSCs culture, indicating DHA was released and functioned when added into culture medium (Figure 6F, G).

**Figure 7:**
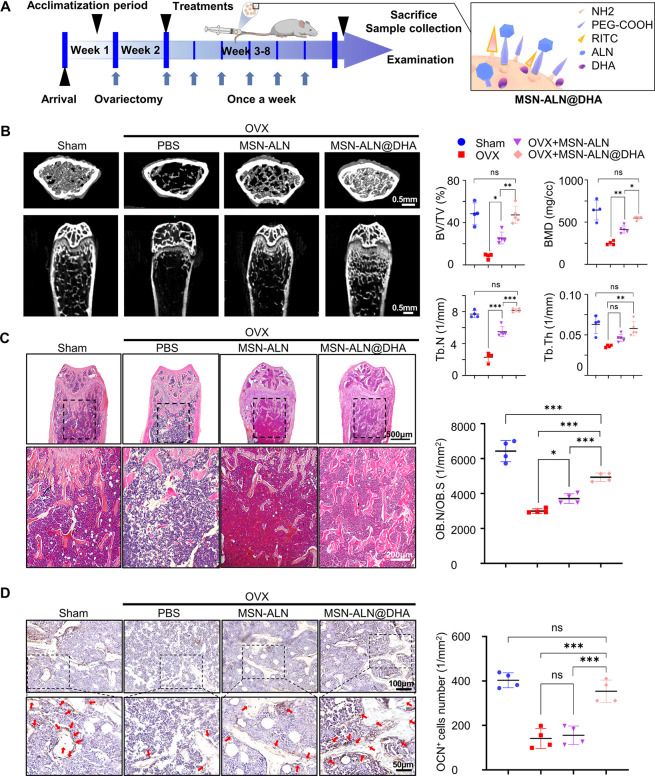
MSN-ALN@DHA promotes bone formation in OVX-induced osteoporotic mice. (A) Schematic illustration of the design of animal experiments. (B) Micro CT of reconstructed 3D images of bone tissues from distal femoral metaphyseal after 6-week treatment and bone morphometric parameters (*n* = 4–5). (C) HE staining of femora from different groups and semiquantitative analysis of OB.N/B.S (*n* = 4). (D) Immunohistochemical staining and semiquantification of OCN^+^ cells (red arrow) in femur bone marrow after MSN-ALN@DHA treatment (*n* = 4). Data were represented as mean ± SD, the *P* values were calculated by one-way ANOVA with Tukey as a posthoc test, and the statistical significance was defined as ***P < 0.001, **P < 0.01, and *P < 0.05 among different groups.

We further verified the efficacy of MSN-ALN@DHA in the treatment of osteoporosis in mice (Figure 7A). Unlike the gavage model, MSN-ALN@DHA was administered weekly via tail vein injection at a dose of 20 mg/kg (equal to 5 mg/kg DHA per week) to OVX mice (MSN-ALN@DHA group). Equivalent amounts of MSN-ALN and saline were applied to the MSN-ALN group; similarly, saline was applied to the OVX-only and sham groups for comparisons. After 8 weeks, Micro CT revealed that MSN-ALN treatment partially alleviated bone loss in the distal femur caused by estrogen deficiency, evidenced by increased BV/TV from 8.30 ± 2.32% to 25.48 ± 5.61%, Tb.Th from 0.036 ± 0.002 mm to 0.047 ± 0.005 mm, Tb.N from 2.29 ± 0.60 to 5.48 ± 0.63/mm, and BMD from 250.99 ± 24.37 to 412.88 ± 42.58 mg/cm^3^. Furthermore, MSN-ALN@DHA performed even better by increasing BV/TV to 47.28 ± 8.23%, Tb.Th to 0.057 ± 0.009 mm, Tb.N to 8.16 ± 0.22/mm, and BMD to 547.79 ± 20.61 mg/cm^3^, which represented that MSN-ALN@DHA treated osteoporosis mice obtained complete bone mass retention and intact trabecular structure comparable to the sham group (Figure 7B). HE staining was in high accordance with the morphological data from micro CT, showing trabecular bone interlaced orderly like a nest in the marrow cavity in the MSN-ALN@DHA and sham groups, far better than that in the OVX group (Figure 7C). MSN-ALNs and MSN-ALN@DHA did not show any hepatotoxicity, nephrotoxicity, cardiotoxicity, pulmonary toxicity, or splenic toxicity (Figure S7A). Additionally, analyses of the ratio of osteoblast number to bone surface (OB. N/B. S.) and OCN^+^ cells revealed that treatment with MSN-ALNs did not significantly restore the osteoblast number or osteoblastic activity, whereas MSN-ALN@DHA almost fully recovered osteoblast functions in osteoporotic mice (Figure 7C, D), confirming that MSN-ALN@DHA was capable of restoring bone mass by targeting BMMSCs, the precursor cells of osteoblasts. Since ALN and DHA have been reported to impede osteoclast activity,^45,46^ tartrate-resistant acid phosphatase (TRAP) staining was used to detect other possible therapeutic targets of MSN-ALN@DHA. Results showed that TRAP^+^ osteoclasts were widely distributed in the epiphysis and metaphysis of femora in the OVX group. MSN-ALN treatment reduced the number of TRAP^+^ osteoclasts to some extent, and MSN-ALN@DHA treatment further suppressed osteoclastic activity, with a few TRAP^+^ osteoclasts similar to those in the sham group (Figure S7B).

Taken together, the therapeutic effect of MSN-ALN@DHA on osteoporosis was mainly achieved by the protection effect of DHA on the stemness of BMMSCs, while both MSN-ALN and DHA also played a certain role in inhibiting osteoclastic activity. The use of the bone-targeting carrier, MSN-ALN, has improved the therapeutic efficacy of DHA. Compared to oral administration of DHA, the application of MSN-ALN@DHA ensures treatment efficacy while reducing the frequency of drug administration.

## Discussion

Osteoporosis is a common bone metabolic disorder that causes severe hip and vertebral fractures and high age-related morbidity, resulting in a heavy socioeconomic burden worldwide.^47^ During osteoporosis progression, the population and functions of osteoblasts are severely attenuated,^48^ leading to disequilibrium between bone formation and bone resorption.^2^ To identify the small molecule compound that can specifically rescue BMMSC stemness from transcriptional profiles, we applied a DLEPS and matched it with DEGs in bone tissues of neonatal mice compared to adult mice and finally identified DHA as a potential candidate. The effect of DHA in promoting BMMSC stemness, stimulating cell proliferation, and maintaining the multipotent differentiation ability during long-term cell expansion was validated in extensive experiments. These findings also demonstrate the potential of deep learning approaches to accelerate drug development and facilitate precision medicine. It is worth noting that DHA is a derivate from traditional Chinese medicine extracts of artemisinin. Since it has been widely used in antimalarial treatment and has been extensively studied over the past four decades for its pharmacological effects, including antimalaria, antitumor invasion, and immunoregulation,^49−52^ its biological security has been guaranteed by clinical practice.^53^ Both DHA and other artemisinin derivates were reported to inhibit osteoclastogenesis by suppressing NF-κB/RankL related pathway in bone regeneration,^45,49,51^ and DHA was also reported to inhibit adipogenesis during 3T3-L1 differentiation,^54^ which added extra relevance of DHA usage in osteoporosis and forecast the broad application and potential value of DHA in the future pharmaceutical market.

Additionally, we elucidated the mechanism of stemness promotion induced by DHA at the genetic level. In general, the acetylation of lysine 9 on histone H3 occurs at the promoters of actively transcribed genes, whereas silenced genes are deacetylated. The H3K9 acetylation level in BMMSCs is crucial for their physiological functions, including proliferation and differentiation. Previous studies have found that during BMMSC fate determination, the H3K9 acetylation level of BMMSC is regulated by both the HATs including PCAF and GCN5 and HDACs, and the upregulation of GCN5 facilitates osteogenesis.^35,36,55,56^ Furthermore, the importance of GCN5 in periodontitis and angiogenesis during osteoporosis has been confirmed in animal disease models.^57^ To screen for the candidate upstream genes that DHA acts on, we performed RT-qPCR to test the expression levels of the 7 H3K9 acetylation-related HATs/HDACs including GCN5, P200, PCAF, SIRT6, HDAC1, HDAC2, and HDAC8. Among the three HATs and four HDACs, the expression of GCN5 showed the most significant change. GCN5, in turn, facilitated the acetylation of Histone 3 lysine 9, leading to the activation of gene expression. Continuous DHA stimulation upregulated stemness markers via the GCN5-H3K9ac axis, whereas the knockdown of GCN5 by siRNA interference counteracted this effect. Chromatin immunoprecipitation would provide further insights into whether acetylation occurs exactly on H3K9 around *SOX2*/ *OCT4* gene promoters, which will be performed in our subsequent research.

Appropriate drug administration methods can improve the treatment efficacy. Given that artemisinin is orally administered in traditional antimalarial practices, we initially chose oral administration to verify the therapeutic effect of DHA on osteoporosis. After the bone restorative efficacy was demonstrated in the gavage model, we created an MSN-ALN@DHA delivery system to make DHA more applicable in osteoporosis treatment. MSNs have attracted interest owing to their excellent properties for drug delivery, such as large surface area, excellent loading capacity owing to their mesoporous architecture, high plasticity of modification, and ensured biocompatibility.^58^ In addition, bisphosphonates with bone-binding affinities such as ALN can absorb onto the bone surface.^43^ Hence, ALN was conjugated to MSN as a bone-targeting ligand for the controlled delivery and release of DHA, which improved the retention time of DHA in bone tissues and reduced the dosage and frequency of DHA administration. MSN-ALN also exerted a limited antiosteoporotic effect, which may stem from the osteoinductive effect of silicate and the osteoclastic inhibition effect of ALN, consistent with our previous findings.^41,43^ Thus, MSN-ALN and DHA synergistically exhibited osteoprotective effects, restoring the trabecular structure to a level similar to that of the sham group.

In summary, based on the analysis of the transcriptional profiles of neonatal and adult femora, we utilized a deep learning-based efficacy prediction system to discover a potential stemness-enhancing drug, DHA, that could promote BMMSCs’ self-renewal and their osteogenic differentiation potential by histone acetylation. In a mouse OVX-induced osteoporosis model, we devised a bone-targeted MSN-ALN@DHA delivery system and demonstrated that it excellently promoted osteogenesis and attenuated adipogenesis, which possesses a promising translational potential for clinical therapies in bone metabolism-related disease.

## Methods

### Gene Expression Profiling by RNA Sequencing

Gene expression profiling of BMMSCs was performed using RNA Sequencing (RNA-seq). Total RNAs were extracted from the femora of neonatal mice (postnatal day 1) and adult mice (6–8 weeks) using Trizol reagents (Thermo Fisher Scientific), followed by purification with the RNeasy mini kit (Qiagen). RNA sequencing (RNA-seq) was conducted by strand-specific library preparation with mRNA enrichment from BGI Tech Solutions (Hongkong), followed by paired-end sequencing with 100 bp read length on the DNBSEQ platform and generated 20 million clean read pairs per sample, which were then mapped onto the GRCh38.p12. The sequencing data were deposited in the NCBI’s SRA database (BioProject ID: PRJNA1020800, http://www.ncbi.nlm.nih.gov/bioproject/1020800). Expression quantifications, differential expression, and gene set enrichment were analyzed by using the BGI RNA-seq pipeline. DEGs were identified based on Student’s *t*-test. The threshold for up- and downregulated genes was identified when the fold change >2 and *P* < 0.05.

### Drug Efficacy Prediction

The Kolmogorov–Smirnov (KS) test is a common method to evaluate the difference between two distributions, especially for those with different sample volumes. The rank list of 12,328 genes was computed using DLEPS to calculate the changes in transcriptional profiles (CTPs). The enrichment score (ES) for up and down gene sets was defined based on relative rank in the query gene list and that in computed CTPs. The ES score for the up gene set is defined as
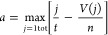
where *t* is the number of genes in the query gene set, *n* is the number of genes in the computed CTPs, and *V*(*j*) is the rank of a specific gene in the rank list. The ES score for the down gene set is defined the same as
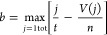


The bone score is defined as
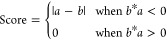


As shown in Figure 1C, the upper right corner shows positively predicted molecules. An FDA-approved library (TargetMol, US, *n* = 961) was used to screen using DEGs as describe in the above paragraph.

### Cell Culture and Expansion

Primary hBMMSCs, purchased from the American Type Culture Collection, were seeded into a 100 mm culture dish (Thermo Fisher Scientific) after cell thawing. They were then incubated in α-modified minimum essential medium (α-MEM; Biological Industries) supplemented with 10% fetal bovine serum (FBS; Thermo Fisher Scientific), 2 mM l-glutamine (Thermo Fisher Scientific), and 100 U/mL penicillin-streptomycin in a humidified atmosphere (37 °C, 5% CO_2_). The fresh medium was changed every 2 days, and hBMMSCs were subcultured at the confluence at 80–90%. DHA dissolved in DMSO was added into culture medium during the passaging process from passage 3 until passage 8. Meanwhile, the vehicle DMSO was applied as a negative control. Both the vehicle-treated and DHA-treated cells were used for subsequent experiments.

Primary mBMMSCs were isolated from the long bones of mice. Specifically, both ends of fresh tibiae and femora were removed, and the bone shaft was flushed with α-MEM. Single-cell suspensions, filtered through a 70-μm strainer, were seeded into culture plastics. Once attached to the disk and formed colony, the mBMMSCs were digested with 0.25% trypsin, reseeded, and subjected to the custom culture procedure mentioned above with α-MEM supplemented with 20% FBS.

### Cell Counting Kit-8 Assay

CCK8 kit (Abcam) was applied to detect cell viability under different DHA concentrations. Following the manual’s instructions, hBMMSCs were plated into 96-well plates (Thermo Fisher Scientific) at a density of 1000 cells per well. After culturing in DHA-containing medium for 0, 1, 3, 5, and 7 days, the plate was incubated with CCK8 working solution for another 2 h. Subsequently, the supernatant absorbance at 450 nm was measured afterward with a spectrophotometer (Bio-Rad).

### Colony-Forming Unit Assay

The colony-forming ability of mBMMSCs was tested by the CFU assay. For each group, 1000 cells were seeded into a 60 mm dish (Thermo Fisher Scientific) and cultured for 10 days. Afterward, the cells were fixed with 4% paraformaldehyde fixation and 1% crystal violet (Solarbio) staining. The number of colonies containing more than 50 cells was counted with ImageJ 1.52v.

### Histological Analysis

The harvested samples thoroughly dissected from soft tissues were immediately fixed in 4% paraformaldehyde overnight. After fixation, decalcification was performed, followed by dehydration using a gradient of alcohol. The samples were then embedded in paraffin blocks for HE staining, Masson’s trichrome staining, TRAP staining, and immunostaining. Alternatively, OCT freezing blocks were used for Oil red O staining. Microtomy was employed to cut the bocks into 5-μm-thick histological sections. HE staining, Masson’s staining, and TRAP staining were performed according to the manufacturer’s (Solarbio) instructions to examine the general appearance of soft tissues and osteoclasts activity, respectively. Stained samples were photographed by a digital camera and a microscope (Leica).

### Immunofluorescence Staining and Immunohistochemistry

Pretreatment for cells on coverslips involved a 1-h-long-PFA fixation and a 10 min-long 0.5% Ttiton X-100 perforation; followed by deparaffination for tissue sections. After blocking nonspecific antigen with 5% BSA for 1 h, samples were incubated overnight at 4 °C with primary antibodies including Ki67 (Abcam), OCN (Abcam), RUNX2 (CST), Mitochondria (Abcam), H3K9ac (Abcam), etc. For immunochemistry staining, horseradish-peroxidase-conjugated secondary antibodies (ZhongShanJinQiao) were applied afterward. For immunofluorescence staining, fluorescein or rhodamine-conjugated secondary antibodies (ZhongShanJinQiao) were applied, and the nuclei were counterstained with 4′,6-diamidino-2-phenylindole (DAPI, Life Technologies). Confocal microscopic images were captured with a laser-scanning microscope (LSM 510, Zeiss, Germany) and processed with LSM 5 Release 4.2. The antibodies involved are listed in the Supporting Information (Table S2)

### Micro CT Analysis of Femora

The femora thoroughly dissected from soft tissue were fixed in 4% PFA overnight after the mice. The samples were then scanned using the Skyscan 1174 micro CT system (Bruker, Belgium) with a resolution of 10.21 μm, a tube voltage of 60 kV and a current of 0.1 mA. The region of interest (ROI) selected for analysis was the diaphysis segment located 0.5 to 1.0 mm below the growth plate in the distal femur. Bone morphometry data of the ROI were obtained by CTAn software.

### Multipotent Differentiation

Osteogenic and adipogenic differentiation was induced in BMMSCs cultured in 12-well plates. Once they reached 70–80% confluence, cells were incubated with differentiation medium. For osteogenic induction, a growth medium supplemented with 10 nM dexamethasone (Sigma-Aldrich), 5 mM β-glycerophosphate (APEXBIO), and 0.05 mM l-ascorbic acid 2-phosphate (Sigma-Aldrich) was applied. Cells samples for RT-qPCR were collected after 7 days of induction, and samples for Western blotting, alkaline phosphatase (ALP) staining after 14 days, and ARS staining after 21 days. The adipogenic differentiation medium consisted of a growth medium supplemented with 500 μM isobutyl-methylxanthine, 60 μM indomethacin, 0.5 μM hydrocortisone, and 10 μM insulin (all form Sigma-Aldrich). Samples for Western blotting and Oil red O assay were collected separately after 14 and 21 days of induction.

### Alkaline Phosphatase Staining

ALP staining and ALP activity determination were performed using the ALP color development kit (Beyotime) and alkaline phosphatase assay kit (Beyotime), respectively, according to the manufacturer’s instructions. Stained samples were photographed by using a digital camera and microscope. For the ALP activity assay, the absorbance of each well was detected with a microplate reader at a wavelength of 520 nm.

### Alizarin Red S Staining

ARS assays were designed to qualitatively and quantitatively analyze extracellular calcium deposits after cell osteogenic induction. The fixed cell plates were incubated with 2% alizarin red S (pH 4.2, Sigma) for 5 min at room temperature. After the floating color was washed away, each well was photographed by a digital camera and a microscope individually. For quantitative analysis, 1 mL of 10% (wt/vol) cetylpyridinium chloride (Sigma) was added to each well to fully dissolve ARS. Afterward, the absorbance of supernatants in each well was detected with a microplate reader at a wavelength of 562 nm.

### Oil Red O Staining

Fixed cell samples or frozen tissue slices were stained with Oil red O assay according to instructions provided in Oil red O staining kit (Solarbio). Different subregions within each well were randomly captured under a microscope, and the Oil red O positive area was determined by ImageJ 5.2v software.

### RT-qPCR Analysis

Total RNA was isolated from cells with a Trizol reagent (Thermo Fisher Scientific) following the manufacturer’s instructions. The isolated mRNA was then converted into complementary DNA (cDNA) using the SuperScript III reverse transcription kit (Invitrogen). Real time PCR was performed using gene-specific primers and SYBR Green (Thermo Fisher Scientific) on a 7500HT Fast Time PCR system. The primers synthesized are given in the Supporting Information (Table S3).

### siRNA Knockdown

To knockdown GCN5 expression in hBMMSCs, siRNA transfection was performed according to the manufacturer’s instructions. A fluorescein-conjugated control siRNA was used as a control to evaluate transfection efficiency. All siRNA products were purchased from the GenePharma Company (China). The targeting sequences for human *GCN5* knockdown were siGCN5-1:5′-GCAUGCCUAAGGAGUAUAUTT-3′ and siGCN5-2:5′-GCUUCACGGAGAUUGUCUUTT-3′.

### Western Blotting

Total proteins from cell lysates were extracted with RIPA buffer (Thermo Fisher Scientific) supplemented with a protease/phosphatase inhibitor cocktail (Thermo Fisher Scientific). Histone samples were prepared with a histone extraction kit (Abcam). The protein samples were separated by Tris-glycine SDS polyacrylamide gel (Thermo Fisher Scientific) and transferred onto a PVDF membrane (EMD Millipore). The membrane was blocked by 5% bovine serum albumin for 1 h at room temperature and incubated overnight at 4 °C with primary antibodies GAPDH, VINCULIN, SOX2, OCT4, OCN, RUNX2, CEBP-α, PPAR-γ, GCN5, Histone 3, and H3K9ac. After washing in Tris-buffered saline-Tween, the membranes were incubated with appropriate secondary antibodies (ZhongShanJinQiao) for 1 h under room temperature. The membranes were thoroughly washed and visualized using a Western Lightning chemiluminescence detection kit (BioRad). Densitometry analysis of the bands was performed with the ImageJ 5.2v software. The antibodies involved are given in the Supporting Information (Table S2).

### Animal Models

Eighty 6-week female C57/6J mice (Charles River) were purchased to establish an ovariectomized osteoporosis model. Additionally, eight 6-week male BALB/c immunocompromised nude mice (Charles River) were obtained for subcutaneous cells implantation. All protocols and procedures were approved by the Animal Use and Care Committee of Peking University (LA2022188).

For subcutaneous transplantation, mineralized collagen scaffolds in the form of cylinders with a diameter of 0.32 cm and a height of 0.1 cm were sterilized with ethylene oxide. The scaffolds were then immersed in single cell suspension for 4 h at 37 °C in an incubator and subsequently implanted subcutaneously. All of the mice were sacrificed after implantation for 8 weeks, and the implants free of soft tissue were excarnated and fixed in 4% PFA.

### Calcein Double Labeling

The mice were intraperitoneally injected with 20 mg/kg of calcein (Sigma-Aldrich) 9 and 2 days prior to euthanasia. Following fixation with 4% PFA and dehydration, the femora were embedded in methyl methacrylate and sectioned into 10 μm slices using a hard tissue slicer. The slices were then observed and photographed under a fluorescence microscope. The distance between two parallel fluorescent deposition lines within the diaphysis was measured to quantify the dynamic bone formation rate over a 7-day period.

### Synthesis and Characterization of MSN-ALN@DHA

For the synthesis of MSN, a one-pot biphase stratification approach with modification was applied to synthesize dendritic MSNs. Tetraethyl orthosilicate (TEOS, Macklin) was used as a silica source, cetyltrimethylammonium chloride (CTAC, Aladdin) as a template, cyclohexane (Macklin) as an emulsion agent, and triethanolamine (TEA, Aladdin) as a catalyst. Specifically, 48 mL of (25 wt %) CTAC solution, 0.36 g of TEA, and 72 mL of deionized water were mixed under stirring in a round-bottom flask at 60 °C for 1 h. Then, 20 mL of (20 v/v%) TEOS solution in cyclohexane was added dropwise to the aqueous phase and the mixture was stirred for another 3 h to form the MSN. Afterward, the MSN products in the water phase were separated with a separating funnel, followed by centrifugation at 36000 g for 1.5 h. Acidic methanol (37% HCl: methanol = 1:10) was employed for MSN purification, and finally, the MSN products were dispersed in ethanol for long-term storage and further functionalization.

For the synthesis of MSN-NH_2_, the MSN was functionalized with amine groups to provide a reactive surface for subsequent covalent conjugation. Briefly, 250 mg of the MSN dispersed in 50 mL of ethanol was supplemented with 1 mL of ammonium hydroxide (28–30%, Sigma) as a catalyst. Then, 4 mL of 3-aminopropyl triethoxysilane (APTES, Sigma) was gently added to react at 25 °C for 24 h. MSN-NH_2_ was extracted by centrifugation and washed in ethanol to remove residual reactants. The MSN-NH_2_ products were dispersed in ethanol for storage and further modification.

For the synthesis of MSN-PEG, 108 mg of *N*-hydroxylsuccinimide-PEG2k-carboxyl (NHS-PEG2k-COOH, Xi’an ruixi Biological Technology Co., Ltd.) was conjugated to 100 mg of MSN-NH_2_ in 25 mL of dimethylformamide (DMF) at 25 °C for 24 h. In addition, 106 mg of NHS-functionalized PEG (NHS-mPEG2k, Xi’an ruixi Biological Technology Co., Ltd.) was used to block residual amine groups. In order to visualize MSN distribution *in vivo*, 2.5 mg of Rhodamine B isothiocyanate (Macklin) was attached by virtue of click-cross-linking between an isothiocyanate group and a remaining amine group in ethanol at 25 °C. The MSN-PEG was dispersed in ethanol for storage and further experiments.

For synthesis of MSN-ALN, ALN was attached onto MSN-PEG by the reaction between the carboxylic group in PEG and the amine group in ALN. Briefly, 10 mg of MSN-PEG dispersed in 10 mL of 0.1 M MES buffer (pH = 6) was mixed with 6.502 mg of ALN. Then, 0.959 mg of *N*-(3-dimethyl aminopropyl)-*N*′-ethylcarbodiimide hydrochloride (EDC, Sigma) and 0.288 mg of *N*-hydroxysuccinimide (NHS, Sigma) were added to catalyze the reaction for 6 h at room temperature. MSN-ALN was washed with deionized water and dispersed in ethanol for further experiment.

For DHA loading, MSN-ALN was dispersed in ethanol with DHA in different feeding ratios (DHA: MSN-ALN = 1:1–4:1). MSN-ALN@DHA was finally acquired after ethanol was thoroughly evaporated under a nitrogen flow. MSN-ALN@DHA was stored at −80 °C in the form of powder.

Surface morphology was presented with SEM, TEM, and DLS. Samples without coating were subjected to SEM and photographed with Hitachi Regulus 8230 scanning electron microscope operated at 5 kV. TEM was performed with a JEOL 1400 transmission electron microscope operated at 100 kV after the nanoparticles were trapped in copper grids. DLS measurements were carried out using a Zetasizer Pro (Malvern Instruments) to analyze particle dispersity and diameter in ethanol, deionized water, and saline, respectively.

The amount of ALN linked to the MSN was estimated by Thermogravimetric analysis (TGA, Mettler Toledo) in the temperature range from 20 to 900 °C at a heating rate of 10 °C/min in air. Ten milligrams of the MSN-PEG and the MSN-ALN milligrams of the MSN-PEG and the MSN-ALN used for the TGA measurement separately.

### Cell Viability

Cell culture tests were performed using hBMMSCs. The cells were seeded in 12-well plates and incubated with varying concentrations of MSN-ALN for 12, 24, and 48 h. Wells without nanoparticles were used as control samples. The viability of the cells was assessed using a live and dead assay kit (Solarbio) following the manufacturer’s instructions. Fluorescent images of the live and dead cells were captured using a laser-scanning microscope.

### MSN-ALN Distribution Tracing in Vivo

To investigate the *in vivo* distribution of nanoparticles, 100 μL of Rhodamine B labeled MSN-ALN or MSN-PEG (2 mg/mL) was injected via tail vein at various time points. Mice were sacrificed at 1 h, 1 day, 4 days, and 7 days postinjection. The intact livers, spleens, femora, and tibiae were dissected from the mice and examined under fluorescence microscopy (PerkinElmer). The distribution of nanoparticles was visualized and analyzed using Caliper IVIS software.

### Statistical Analysis

All data were reported as mean ± SD. GraphPad Prism software, version 8.0.2, was applied for statistical analyses. The significance of differences was determined by unpaired Student’s *t*-test or one-way ANOVA with Tukey’s post hoc test. Differences were considered statically significant when *P* < 0.05. The presented data were obtained from a minimum of three independent experiments.

### Safety Statement

No unexpected or unusually high safety hazards were encountered.

## Supporting Information Available

Figure S1. The representative 3D micro CT reconstructed images demonstrating the alleviation in height loss of the mesial alveolar bone of the 1st molar in osteoporotic mice after 4 weeks of oral administration with DHA. Figure S2. HE staining of liver and kidney showing no *in vivo* toxicity of DHA oral administration under current dose. Figure S3. Western blotting of H3K9ac in hBMMSCs at early and late passages. Figure S4. Functional validation of small interfering RNA siGCN5. Figure S5. RT-qPCR and Western blotting of *SOX2* and *OCT4* in hBMMSCs with GCN5 knockdown followed by DHA treatment. Figure S6. Synthesis process and characterization of MSN-ALN@DHA. Figure S7. (A) HE staining of the major organs after intravascular injection of MSN-ALN@DHA exhibiting no *in vivo* toxicity. (B) TRAP staining of femur trabecular bone from different groups and semiquantitative analysis of TRAP^+^ cell number per unit area (*n* = 4). Figures S8–S9. Original scans of the blots in figures. Table S1. List of top-ranked molecules by DLEPS. Table S2. List of primary and secondary antibodies used in the study. Table S3. List of primers used in the study. Table S4. List of chemicals and commercial assays used in the study. Table S5. Software and algorithms (PDF)
