# Supplementary material for: Deep Learning-Predicted Dihydroartemisinin Rescues Osteoporosis by Maintaining Mesenchymal Stem Cell Stemness through Activating Histone 3 Lys 9 Acetylation
Source: ACS Cent Sci. 2023 Oct 18;9(10):1927–43. doi: 10.1021/acscentsci.3c00794 (PMC10604014; doi:10.1021/acscentsci.3c00794)
Supplement: Supplementary file 1 — oc3c00794_si_001.pdf [file oc3c00794_si_001.pdf]

Deep learning-predicted dihydroarteminsinin rescues osteoporosis by maintaining mesenchymal stem cell stemness through activating Histone3 Lys9 acetylation

Ruoxi Wang[†,#], Yu Wang[†,#], Yuting Niu [‡,#], Danqing He[†], Shanshan Jin[†], Zixin Li[†], Lisha Zhu[†], Liyuan Chen[†], Xiaolan Wu[†], Chengye Ding[†], Tianhao Wu[†], Xinmeng Shi[†], He Zhang[†], Chang Li[†], Xin Wang[Δ], Zhengwei Xie[Δ,*], Weiran Li[†,*], Yan Liu[†,*]

[†]Central Laboratory, Peking University School and Hospital for Stomatology & National Center for Stomatology & Laboratory of Biomimetic Nanomaterials, Department of Orthodontics, Peking University School and Hospital for Stomatology & National Center for Stomatology & National Clinical Research Center for Oral Diseases & National Engineering Laboratory for Digital and Material Technology of Stomatology & Beijing Key Laboratory of Digital Stomatology & Research Center of Engineering and Technology for Computerized Dentistry Ministry of Health & NMPA Key Laboratory for Dental Materials & Translational Research Center for Orocraniofacial Stem Cells and Systemic Health, Beijing 100081, China

[‡]Central Laboratory, Peking University School and Hospital for Stomatology & National Center for Stomatology & National Clinical Research Center for Oral Diseases & National Engineering Laboratory for Digital and Material Technology of Stomatology & Beijing Key Laboratory of Digital Stomatology & Research Center of Engineering and Technology for Computerized Dentistry Ministry of Health & NMPA Key Laboratory for Dental Materials & Translational Research Center for Orocraniofacial Stem Cells and Systemic Health, Beijing 100081, China

[Δ]Peking University International Cancer Institute, Health Science Center, Peking University, Beijing 100083, China

[#]These authors contributed equally: Ruoxi Wang, Yu Wang, Yuting Niu

*These authors jointly supervised this work: Zhengwei Xie, Weiran Li, Yan Liu

*Email: orthoyan@bjmu.edu.cn.

Supporting Information

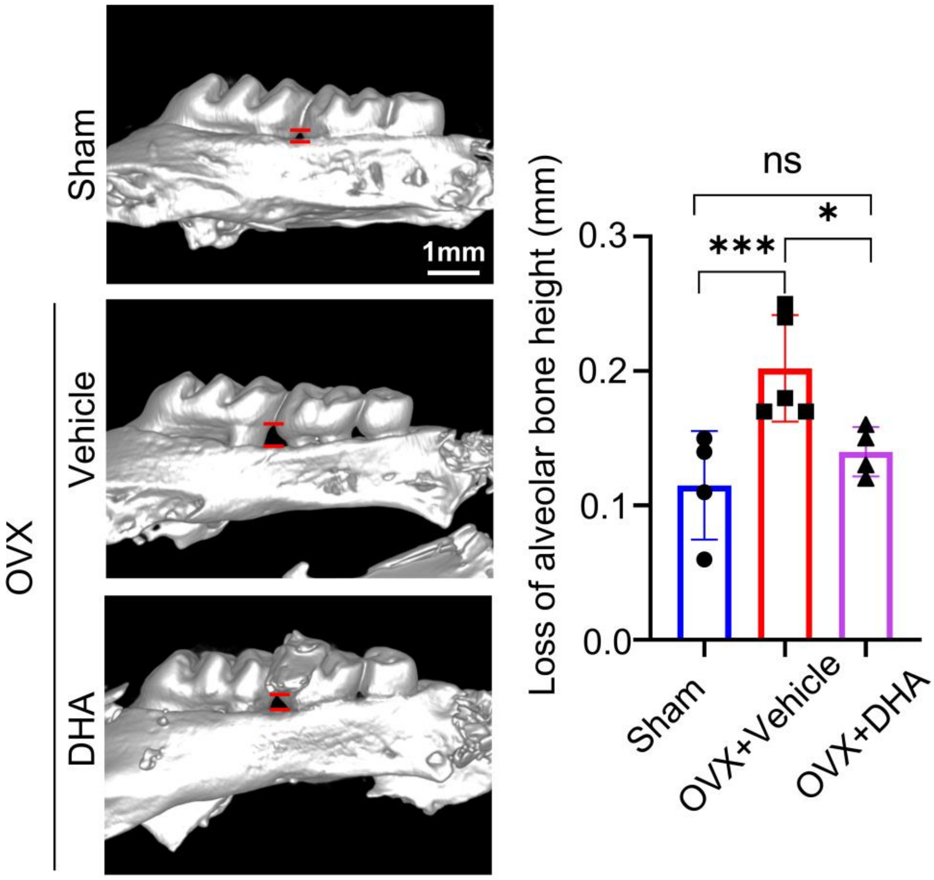

Figure S1. The representative 3D micro CT reconstruction images demonstrate the alleviation in height loss of the mesial alveolar bone of the 1st molar in osteoporotic mice after 4 weeks of oral treatment with DHA. The alveolar bone height loss was labelled by spacing between red segments ($n = 4$). Data were represented as mean ± SD and the P values were calculated by one-way ANOVA with Tukey as post-hoc test, and the statistical significance was defined as $^{***}P < 0.001$ and $^{*}P < 0.05$ among different groups.

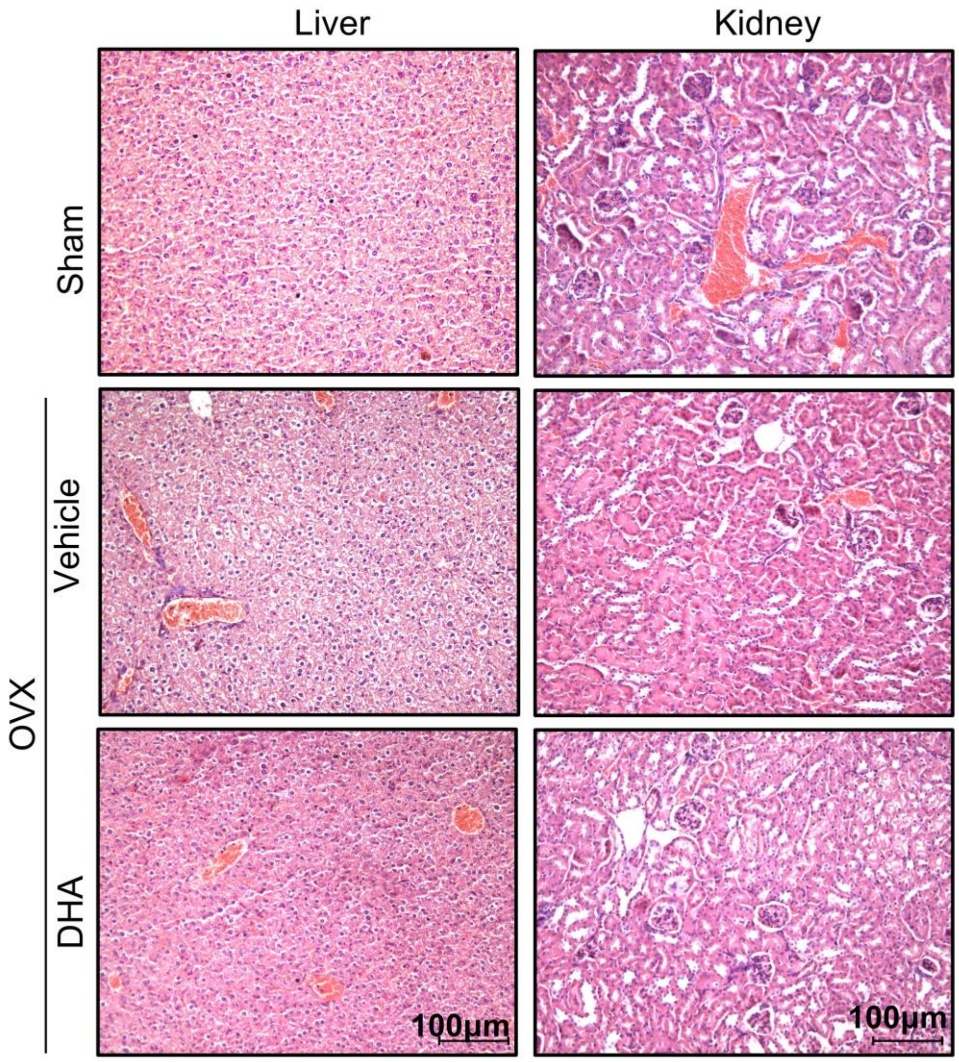

Figure S2. HE staining of liver and kidney shows no *in vivo* toxicity of DHA during oral administration under current dose.

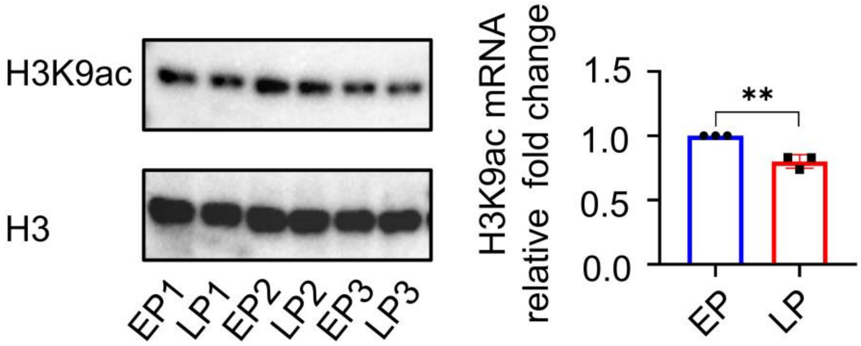

Figure S3. Western blotting of H3K9ac in hBMMSCs at early and late passages (n = 3). EP: early passages; LP: late passages. Data were represented as mean ± SD and the

P values were calculated by two-tailed Student's *t* test. **$P < 0.01$.

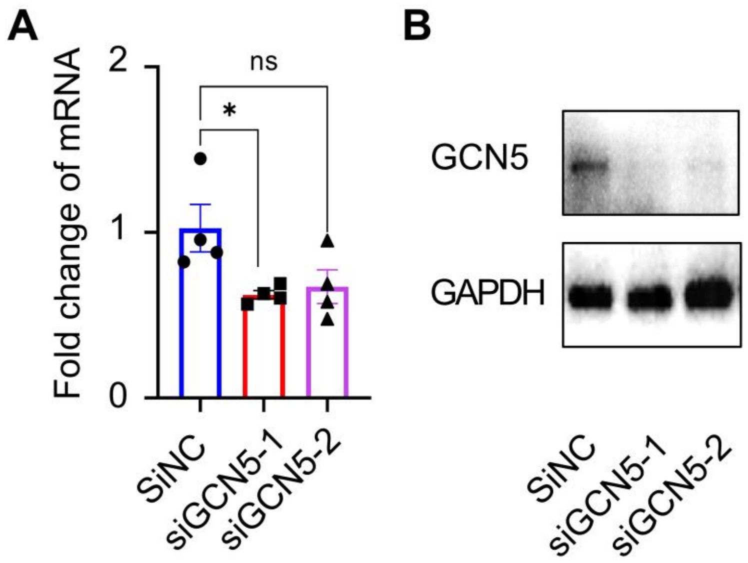

Figure S4. Functional validation of small interfering RNA siGCN5. (A) RT-qPCR of *GCN5* in hBMMSCs after knocking down by small interfering RNA. siNC: negative control siRNA (n = 3). Data were represented as mean ± SD and the *P* values were calculated by two-tailed Student's *t* test. *$P < 0.05$. (B) Western blotting and semi-quantification of GCN5 in siNC- and siGCN5-treated hBMMSCs.

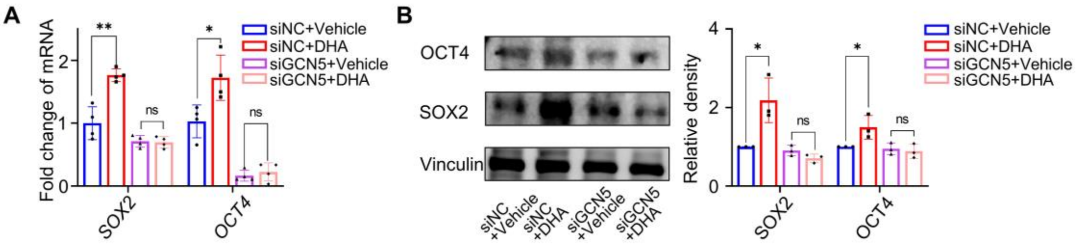

Figure S5. (A) RT-qPCR of *SOX2* and *OCT4* in hBMMSCs with GCN5 knockdown followed by DHA treatment (n = 4). (B) Western blotting of SOX2 and OCT4 in hBMMSCs with GCN5 knockdown followed by DHA treatment (n = 3).

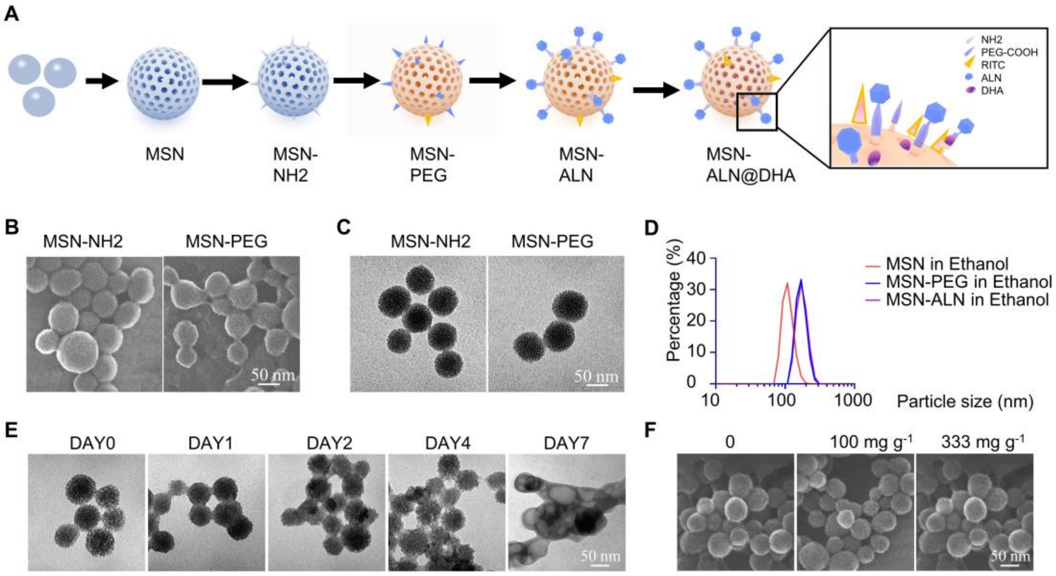

Figure S6. Synthesis process and characterization of MSN-ALN@DHA. (A) Schematic illustration of the procedure to fabricate MSN-ALN@DHA, specifically MSN synthesis via Stöber method followed by -NH_2 functionalization and ALN conjugation. (B-C) SEM and TEM images of intermediate products MSN-NH_2 and MSN-PEG. (D) DLS plot showing diameters distribution of MSNs, MSN-NH_2 and MSN-PEG in ethanol. (E) TEM images of degraded MSN-ALNs after incubated with stirred 37°C PBS for 1, 2, 4, and 7 days. (F) SEM images of MSN-ALN loading with DHA at different proportions.

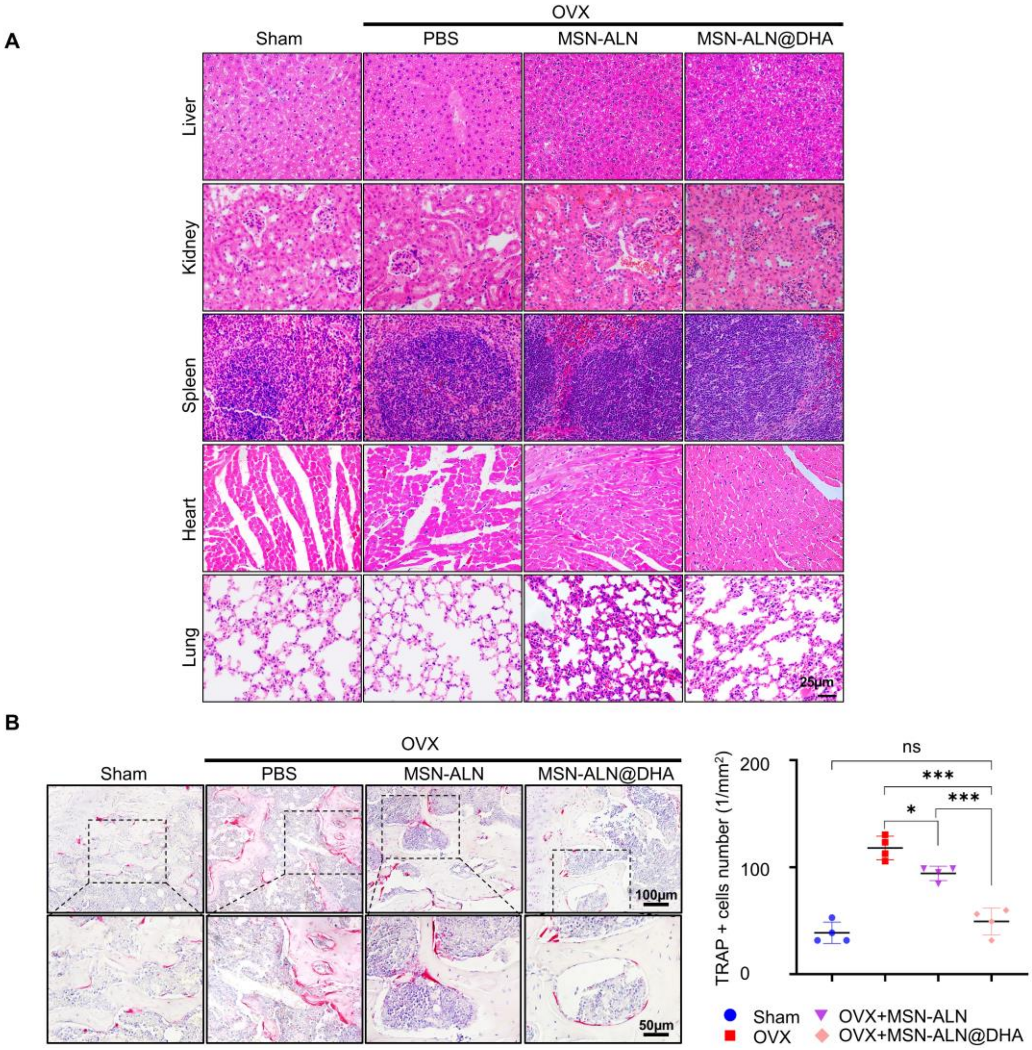

Figure S7. (A) HE staining of the major organs after intravascular injection of MSN-ALN@DHA exhibits no *in vivo* toxicity. (B) TRAP staining of femur trabecular bone from different groups and semiquantitative analysis of TRAP$^+$ cells number per unit area (n = 4). Data were represented as mean ± SD and the P values were calculated by one-way ANOVA with Tukey as post-hoc test, and the statistical significance was defined as ***P < 0.001 and *P < 0.05 among different groups.

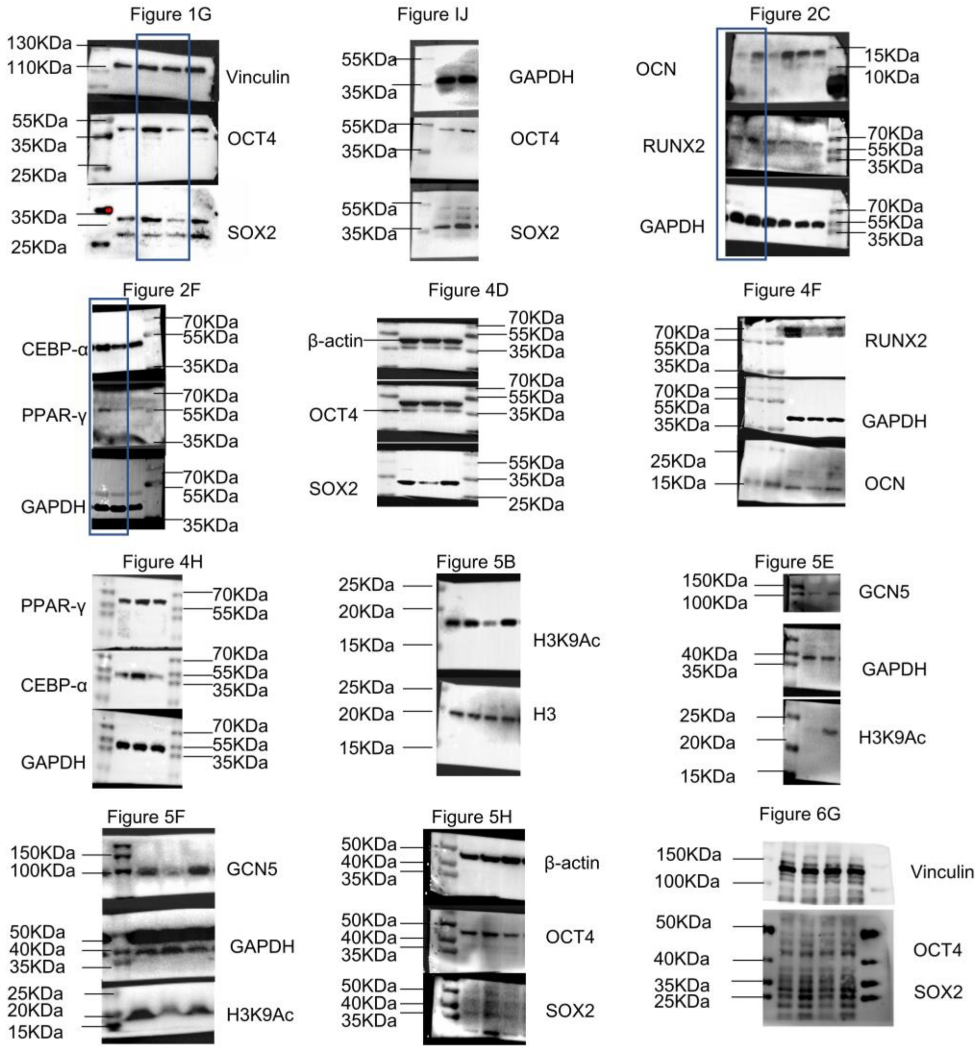

Figure S8. Original scans of the blots in Figure 1, 2, 4, 5 and 6.

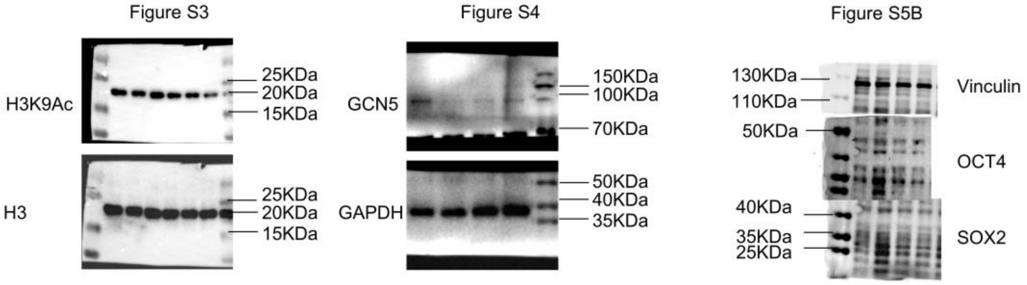

Figure S9. Original scans of the blots in Figure S3-5.

Table S1. List of top-ranked molecules by DLEPS.

Index	ID	Name	CAS	cs_mice_bone
423	S2290	**Dihydroartemisinin (DHA)**	71939-50-9	0.33927
136	S1408	Linezolid	165800-03-3	0.32095
420	S2264	Artemether	71963-77-4	0.29459
177	S1565	VX-809 (Lumacaftor)	936727-05-8	0.28614
352	S1964	Rimantadine	13392-28-4	0.27952
931	S1370	Biapenem	120410-24-4	0.27804
440	S2461	Domperidone	57808-66-9	0.27675
755	S4584	Butylparaben	94-26-8	0.27479
612	S4054	Spironolactone	52-01-7	0.27382
135	S1407	Bimatoprost	155206-00-1	0.26391

Table S2. List of primary and secondary antibodies used in the study.

Antibodies	Source	Identifier	Dilution
Rabbit-polyclonal anti-Sox2	Abcam	AB97959	WB (1:1000)
Rabbit-polyclonal anti-Oct4	Abcam	AB181557	WB (1:1000)
Rabbit-monoclonal anti-Ki67	Abcam	AB16667	IF of tissue (1:200), IF of cell (1:400)
Rabbit-polyclonal anti-Osteocalcin	Proteintech	23418-1-AP	IF of tissue (1:200), WB (1:1000)
Rabbit-polyclonal anti-Osx/ Sp7	Abcam	AB 209484	IF of tissue (1:200)
Mouse-monoclonal anti-Runx2	Abcam	AB236639	WB (1:1000)
Rabbit-monoclonal anti-Cebp-α	Abcam	AB40764	WB (1:1000)
Rabbit-polyclonal anti-Ppar-γ	Abcam	AB209350	WB (1:1000)
Rabbit-monoclonal anti-Fabp4	Abcam	AB92301	IF of tissue (1:200)
Mouse-monoclonal anti-Mitochondria	Abcam	AB 92824	IF of tissue (1:200)
Rabbit-monoclonal anti-Histone H3 (acetyl K9)	Abcam	AB32129	IF of tissue (1:200), IF of cell (1:400), WB (1:1000)
Mouse-monoclonal anti-Kat2a / Gcn5	Abcam	AB208097	WB (1:1000)
Mouse anti-Actin	ZSGB-BIO	TA-09	WB (1:3000)
Mouse-monoclonal anti-	Proteintech	66305-1-lg	WB (1:3000)

Vinculin			
Rabbit-monoclonal anti-Gapdh	Cell Signaling Technology	#5174	WB (1:3000)
Mouse-monoclonal anti-Histone H3	Santa cruz	Sc-517576	WB (1:1000)
HRP-linked anti-mouse IgG	Cell Signaling Technology	7076S	WB (1:10000)
HRP-linked anti-rabbit IgG	Cell Signaling Technology	7074S	WB (1:10000)
FITC-labeled goat anti-mouse IgG (H + L)	ZSGB-BIO	ZF-0312	IF (1:300)
FITC-labeled goat anti-rabbit IgG (H + L)	ZSGB-BIO	ZF-0311	IF (1:300)
Rhodamine labeled goat anti-mouse IgG (H + L)	ZSGB-BIO	ZF-0313	IF (1:300)
Rhodamine labeled goat anti-rabbit IgG (H + L)	ZSGB-BIO	ZF-0316	IF (1:300)

Table S3. List of primers used in the study.

Gene	Primer sequence (5’ - 3’)	
h-*SOX2*	FORWARD	GCTACAGCATGATGATGCAGGACCA
	REVERSE	TCTGCGAGCTGGTCATGGAGTT
h-*OCT4/ POU5F1*	FORWARD	CCTGAAGCAGAAGAGGATCACC
	REVERSE	AAAGCGGCAGATGGTCGTTTGG
h-*GAPDH*	FORWARD	AATCCCATCACCATCTTCCAG
	REVERSE	GAGCCCCAGCCTTCTCCAT
h-*OSX/ SP7*	FORWARD	AAAGGAGGCACAAAGAAGC
	REVERSE	CAGGAAATGAGTGAGGGAAG
h-*RUNX2*	FORWARD	TAGGCGCATTTCAGGTGCTT
	REVERSE	GGTGTGGTAGTGAGTGGTGG
h-*OCN*	FORWARD	AAATAGCCCTGGCAGATTCC
	REVERSE	CAGCCTCCAGCACTGTTTAT
h-*GCN5*	FORWARD	CTAGGGGTCTTCTCGGCTTG
	REVERSE	GGATACGTGGTCAGCCAAGG
h-*P300*	FORWARD	GCAGTGTGCCAAACCAGATG
	REVERSE	CATAGCCCATAGGCGGGTTG
h-*PCAF*	FORWARD	CTGCAAGGCCGAGGAGTCT
	REVERSE	GAAACATGAGCAGCTAGGGC
h-*SIRT6*	FORWARD	CCCCGACTTCAGGGGTCC
	REVERSE	TTCTGGCTGACCAGGAAGC
h-*HDAC1*	FORWARD	ACGACGGGGATGTTGGAAAT
	REVERSE	TGGCTTTGTGAGGGCGATAG

h-*HDAC2*	FORWARD	GGGACTATCGCCCCCAC
	REVERSE	ATAATTTCCAATATCACCGTCGTAG
h-*HDAC8*	FORWARD	ACTCCATAGAATATGGGCTAGGTTA
	REVERSE	AGGAAAGAGTCAGAAAACAGAAAGG
ms-*Sox2*	FORWARD	CAGGAGTTGTCAAGGCAGAGA
	REVERSE	CGCCGCGATTGTTGTGATTA
ms-*Oct4*	FORWARD	CGGAAGAGAAAGCGAACTAGC
	REVERSE	ATTGGCGATGTGAGTGATCTG
ms-*Gapdh*	FORWARD	ATACGGCTACAGCAACAGGG
	REVERSE	TGTGAGGGAGATGCTCAGTG
ms-*Runx2*	FORWARD	TGGTTACTGTCATGGCGGGTA
	REVERSE	TCTCAGATCGTTGAACCTTGCTA
ms-*Ocn*	FORWARD	ATGAGCCCTCAGACTCCTC
	REVERSE	CGGCCGTAGAGCGCCGATA
ms-*Alp*	FORWARD	CCAACTCTTTTGTGCCAGAGA
	REVERSE	GGCTACATTGGTGTTGAGCTT TT

Table S4. List of chemicals and commercial assays used in the study.

Chemicals, peptides, and recombinant proteins		
Dyhydroartemisinin	Selleck	Cat#S2290
β-Glycerophosphate	Selleck	Cat#S3620
Insulin	Sigma-Aldrich	Cat#10516
IBMX	Selleck	Cat#S5836
Indomethacin	Sigma-Aldrich	Cat#53-86-1
Penicillin-Streptomycin	Thermo Fisher Scientific	Cat#15070063
Trypsin-EDTA	Hyclone	Cat#SH30042.01
TRIzol Reagent	Thermo Fisher Scientific	Cat#15596026
Crystal Violet Stain solution	Solarbio	Cat#G1062
Collagen I, Rat Tail	Corning	Cat#54236
RNeasy mini Kit	Qiagen	Cat#74104
Mounting Medium with DAPI	ZSGB-BIO	Cat#ZLI-9557
Dimethyl sulfoxide	Solarbio	Cat#D8371
Phosphate buffered solution	Solarbio	Cat#P1020
RIPA Buffer	Thermo Fisher Scientific	Cat#89900
L-Glutamine	Thermo Fisher Scientific	Cat#25030081
L-Ascorbic acid	Sigma-Aldrich	Cat#A5960
SYBR Green Supermix	Thermo Fisher Scientific	Cat#4385612
Fetal bovine serum (FBS)	Thermo Fisher Scientific	Cat#10099-141
Alizarin Red S	Sigma-Aldrich	Cat#A5533
α-MEM	BI	Cat# 01-043-1A
Dexamethasone	Sigma-Aldrich	Cat#D8893
Lyso-Tracker Red probe	Beyotime	Cat# C1046

TBS	Solarbio	T1080
Tween	Sigma-Aldrich	P9416
Critical commercial assays		
ReverTra Ace qPCR RT Kit	TOYOBO	Cat#FSQ-101
Oil Red O stain Kit	Solarbio	Cat#G1262
BCIP/NBT Alkaline phosphatase color development Kit	Beyotime	Cat#C3206
Alkaline phosphatase assay Kit	Beyotime	Cat#P0321S
Tartrate-resistant acid phosphatase (TRAP) staining Kit	Solarbio	Cat#G1050
HE staining Kit	Solarbio	Cat#G1005
Pierce BCA protein assay Kit	Thermo Fisher Scientific	Cat#23225
Enhanced chemiluminescence western blotting detection Kit	Thermo Fisher Scientific	Cat#34577
Histone extraction Kit	Abacam	Cat#ab112476

Table S5. Software and Algorithms.

Gene ontology	https://geneontology.org/
DLEPS	This paper
GSEA 3.0	http://software.broadinstitute.org/gsea/index.jsp
μCT Evaluation CTAn software	https://www.blut-scientific.com/bruker-micro-ct-software/
Graph Pad Prism 8.0	https://www.graphpad.com/
IVIS Spectrum In Vivo Imaging System (PerkinElmer)	https://www.perkinelmer.com/Product/ivis-spectrum-imaging-system
Nanoscope analysis 1.9	https://www.bruker.com/
ImageJ/Fiji	http://fiji.sc
